# Discovery of Two β-1,2-Mannoside Phosphorylases Showing Different Chain-Length Specificities from *Thermoanaerobacter* sp. X-514

**DOI:** 10.1371/journal.pone.0114882

**Published:** 2014-12-12

**Authors:** Kazuhiro Chiku, Takanori Nihira, Erika Suzuki, Mamoru Nishimoto, Motomitsu Kitaoka, Ken'ichi Ohtsubo, Hiroyuki Nakai

**Affiliations:** 1 Faculty of Agriculture, Niigata University, Niigata, Japan; 2 National Food Research Institute, National Agriculture and Food Research Organization, Tsukuba, Ibaraki, Japan; INRA, France

## Abstract

We characterized Teth514_1788 and Teth514_1789, belonging to glycoside hydrolase family 130, from *Thermoanaerobacter* sp. X-514. These two enzymes catalyzed the synthesis of 1,2-β-oligomannan using β-1,2-mannobiose and d-mannose as the optimal acceptors, respectively, in the presence of the donor α-d-mannose 1-phosphate. Kinetic analysis of the phosphorolytic reaction toward 1,2-β-oligomannan revealed that these enzymes followed a typical sequential Bi Bi mechanism. The kinetic parameters of the phosphorolysis of 1,2-β-oligomannan indicate that Teth514_1788 and Teth514_1789 prefer 1,2-β-oligomannans containing a DP ≥3 and β-1,2-Man_2_, respectively. These results indicate that the two enzymes are novel inverting phosphorylases that exhibit distinct chain-length specificities toward 1,2-β-oligomannan. Here, we propose 1,2-β-oligomannan:phosphate α-d-mannosyltransferase as the systematic name and 1,2-β-oligomannan phosphorylase as the short name for Teth514_1788 and β-1,2-mannobiose:phosphate α-d-mannosyltransferase as the systematic name and β-1,2-mannobiose phosphorylase as the short name for Teth514_1789.

## Introduction

Glycoside phosphorylases catalyze the cleavage of glycosyl linkages via a substitution with inorganic phosphate [1]–[4]. These enzymes phosphorolyze particular glycosides to form corresponding sugar 1-phosphates with retention or inversion of the anomeric configuration [1]–[4]. Because the phosphorylase reactions are reversible, various oligosaccharides have been synthesized via reverse phosphorolysis using the corresponding sugar 1-phosphate as a donor substrate and suitable carbohydrate acceptors [3], [4]. In addition, these reversible catalytic reactions are well suited for the practical synthesis of oligosaccharides from abundantly available natural sugars without using costly sugar 1-phosphate as the starting material by using a single phosphorylase [1], [3], [5] or by combined reaction with two phosphorylases that share the same sugar 1-phosphate [6]–[8] or that produce different sugar 1-phosphates with additional enzymes to convert the sugar 1-phosphates [8]–[10]. However, the relatively narrow range of variations of phosphorylases limits the application of these phosphorylases. Therefore, the discovery of a novel phosphorylase showing unreported substrate specificity and regioselectivity is desired to expand the number of synthesizable oligosaccharides.

Phosphorylases have been classified as members of glycoside hydrolase families (GH) 13, 65, 94, 112, and 130 and glycosyltransferase families 4 and 35 in the Carbohydrate-Active Enzymes database (http://www.cazy.org/) based on their amino acid sequence similarities [11]. Among these families, GH130 is composed of phosphorylases that catalyze the reversible phosphorolysis of β-mannosides to form α-d-mannose 1-phosphate (α-Man1*P*) with an inversion of its anomeric configuration. The stereoselective configurations of β-mannosides are a considerable challenge in synthetic glycochemistry because the vicinal C2 hydroxyl group blocks access to the β-face due to its steric and polar effects [12]. Therefore, a reverse phosphorolysis with strict regioselectivity can be a strong tool for the efficient preparation of β-mannosides. Currently, four phosphorylases, 4-*O*-β-mannosyl-d-glucose phosphorylase (EC 2.4.1.281) [13]–[15], β-1,4-mannooligosaccharide phosphorylase (EC 2.4.1.319) [14], 1,4-β-mannosyl-*N*-acetyl-d-glucosamine phosphorylase (EC 2.4.1.320) [10], and β-mannopyranosyl-[*N*-glycan] phosphorylase (EC 2.4.1.-) [16], are categorized into GH130.

Currently, all of the reported GH130 phosphorylases have originated from anaerobes and are considered to be involved in the catabolism of β-mannosides under anaerobic conditions. In *Ruminococcus albus*, a 4-*O*-β-mannosyl-d-glucose phosphorylase and a β-1,4-mannooligosaccharide phosphorylase have been proposed to be involved in the degradation of the hemicellulosic β-1,4-mannan in place cells, together with GH26 *endo*- and/or *exo*-β-mannanases (EC 3.2.1.78 and EC 3.2.1.-) and a cellobiose 2-epimerase (EC 5.1.3.11) [14]. In *Bacteroides thetaiotaomicron*, a GH130 1,4-β-mannosyl-*N*-acetyl-d-glucosamine phosphorylase releases α-Man1*P* from a common core disaccharide of *N*-glycans that have been liberated by sequential glycoside hydrolase-catalyzed reactions from a complex-type *N*-glycan [10]. The resultant α-Man1*P* is converted into d-fructose 6-phosphate from Man6*P* via the sequential reaction of phosphomannomutase (EC 5.4.2.8) and mannose-6-phosphate isomerase (EC 5.3.1.8) and enters glycolysis. The catabolic pathways that include GH130 phosphorylases that enable anaerobes to produce α-Man1*P* directly without consuming ATP are energetically efficient when compared with the conventional catabolic pathway that contains ATP-dependent carbohydrate kinase because only three molecules of ATP are available via the glycolytic pathway from glucose 6-phosphate.

In this study, we noticed that the anaerobic thermophile *Thermoanaerobacter* sp. X-514 possesses two genes encoding two GH130 proteins, Teth514_1788 and Teth514_1789, which have unknown functions in the genome. Here, we describe two novel GH130 phosphorylases that show unique substrate specificity toward 1,2-β-oligomannan. Interestingly, our results suggest that both phosphorylases are involved in GDP-d-mannose biosynthesis in this anaerobic bacterium.

## Materials and Methods

### Sequence Analysis

Similarity searches were performed at the Swiss Institute of Bioinformatics using the basic local alignment search tool (BLAST) network service (http://web.expasy.org/blast/). The National Center for Biotechnology Information (NCBI) BLASTP tool was used for searching the Swiss-Prot/TrEMBL database [17]. The protein localization and the signal peptides were predicted using PSORTb Version 3.0.2 (http://www.psort.org/psortb/) [18] and the SignalP 4.1 server (http://www.cbs.dtu.dk/services/SignalP/) [19], respectively.

### Cloning, Expression, and Purification

The two genes encoding Teth514_1788 and Teth514_1789 (GenBank accession numbers ABY93074 and ABY93073, respectively) were amplified from the genomic DNA of *Thermoanaerobacter* sp. X-514 via a PCR performed using KOD-plus DNA polymerase (Toyobo, Osaka, Japan) together with the following oligonucleotides, which were designed based on the genomic sequence (GenBank accession number CP000923) [20]: 5′-ggaattccatatgataaaattaaagagatt-3′ as the forward primer containing an NdeI site (underlined) and 5′-tttctcgagaaatttgatatctttcatctc-3′ as the reverse primer containing an XhoI site (underlined) for Teth514_1788; and 5′-ggaattccatatgttcaggctaacaagact-3′ as the forward primer containing an NdeI site (underlined) and 5′-tttctcgagaaattttactttttctttttc-3′ as the reverse primer containing an XhoI site (underlined) for Teth514_1789. The amplified genes were purified using a FastGene Gel/PCR Extraction Kit (Nippon Genetics Co, Tokyo, Japan), digested with NdeI and XhoI (New England Biolabs, Beverly, MA, USA), and inserted into pET24a (+) (Novagen, Madison, WI, USA) to encode a His_6_-tagged fusion at the C-terminus of each recombinant protein. The expression plasmids were propagated in *Escherichia coli* DH5α (Toyobo), purified using a FastGene Plasmid Mini Kit (Nippon Genetics Co.), and verified via sequencing (Operon Biotechnologies, Tokyo, Japan). An *E. coli* Rosetta 2 (DE3) (Novagen) transformant harboring each of the expression plasmids was grown at 37°C in 200 mL of Luria-Bertani medium (1% tryptone, 0.5% yeast extract, and 0.5% NaCl) containing 50 µg·mL^−1^ kanamycin and 30 µg·mL^−1^ chloramphenicol until the absorbance reached 0.6 at 600 nm. The expression was then induced using 0.1 mm isopropyl-β-d-thiogalactopyranoside and continued at 18°C for 24 h. The cells were harvested via centrifugation at 10,000×*g* for 20 min and suspended in 50 mm HEPES-NaOH buffer (pH 7.0) containing 500 mm NaCl (buffer A). The suspended cells were disrupted via sonication (Branson Sonifier 250A; Branson Ultrasonics Division of Emerson Japan, Kanagawa, Japan), and the supernatant was collected via centrifugation at 20,000×*g* for 20 min. The supernatant was applied to a HisTrap FF column (GE Healthcare, Buckinghamshire, UK) and equilibrated with buffer A containing 10 mm imidazole using an ÄKTA Prime (GE Healthcare). After washing with buffer A containing 22 mm imidazole and subsequently eluting the proteins with a 22–400 mm imidazole linear gradient in buffer A, the fractions containing the recombinant protein were pooled, dialyzed against 10 mm HEPES-NaOH buffer (pH 7.0), and concentrated (AMICON Ultra-15 filter; Millipore, Billerica, MA, USA). The protein concentrations were determined spectrophotometrically at 280 nm using the theoretical extinction coefficients of ε = 65,320 and 78,840 m ^−1^ cm^−1^, which are based on the amino acid sequences of Teth514_1788 and Teth514_1789, respectively [21]. The molecular masses of the proteins were estimated via SDS-PAGE (Mini-PROTEAN Tetra electrophoresis system using 4–15% MiniPROTEAN TGX Precast Polyacrylamide Gels; Bio-Rad Laboratories, Inc., Hercules, CA, USA) using BLUE Star Prestained Protein-Ladder (Nippon Genetics Co.) as standard and gel filtration chromatography (HiLoad 26/600 Superdex 200 pg; GE Healthcare), which was performed using a column equilibrated with 10 mm HEPES-NaOH buffer (pH 7.0) containing 150 mm NaCl; the flow rate was 0.5 ml/min and Marker Proteins for Molecular Weight Determination on High Pressure Liquid Chromatography (Oriental Yeast, Tokyo, Japan) were used as standards.

### Measurement of Synthetic Activity

The synthetic activity was routinely determined by measuring the increase in inorganic phosphate (P_i_) using a reaction mixture containing 10 mm α-Man1*P* (α-Man1*P* bis(cyclohexylammonium) salt, which was synthesized from d-mannose and ATP using *N*-acetylhexosamine 1-kinase [22], [23]) and 10 mm d-mannose in 40 mm sodium acetate buffer (pH 5.0 for Teth514_1788 or pH 5.5 for Teth514_1789) at 30°C, following the method created by Lowry and Lopez [24] as described previously [10].

### Acceptor Specificity Analysis

To investigate the acceptor specificities of Teth514_1788 and Teth514_1789, the synthetic reactions were examined using the standard conditions described in the preceding subsection. In the reactions, we substituted d-mannose with the following putative carbohydrate acceptors: d-altrose, d-fructose, d-glucosamine, d-glucose, isomaltose, kojibiose, lactose, lactulose, maltose, melibiose, methyl-α-d-glucoside, methyl-β-d-glucoside, nigerose, l-rhamnose, d-ribose, sophorose, sucrose, d-talose, trehalose, xylobiose, and d-xylose (Wako Pure Chemicals, Osaka, Japan); *N*-acetyl-d-galactosamine, *N*-acetyl-d-glucosamine, *N*-acetyl-d-mannosamine, d-allose, 1,5-anhydro-d-glucitol, 2-deoxy-d-glucose, d-galactosamine, d-galactose, d-galacturonic acid, gentiobiose, d-lyxose, and 3-*O*-methyl-d-glucose (Sigma-Aldrich, St. Louis, MO, USA); d-arabinose, l-arabinose, cellobiose, and d-glucuronic acid (Tokyo Chemical Industry, Tokyo, Japan); and *N*,*N*′-diacetylchitobiose and laminaribiose (Seikagaku Biobusiness, Tokyo, Japan). The reactions contained 120 µm Teth514_1788 or 22 µm Teth514_1789 and were performed for 2 h at 30°C. The reaction mixtures were spotted on TLC plates (Kieselgel 60 F_254_; Merck, Darmstadt, Germany), and the plates were developed using a mobile phase of 80% acetonitrile in water. The TLC plates were soaked in a 5% sulfuric acid-methanol solution and heated in an oven until bands appeared.

### Donor Specificity Analysis

To investigate the donor specificities of Teth514_1788 and Teth514_1789, the synthetic reactions were examined under the aforementioned standard conditions, and we substituted α-Man1*P* with the following sugar-phosphate derivatives: β-l-fucose 1-phosphate, α-d-galactose 1-phosphate, α-d-glucosamine 1-phosphate, α-d-glucose 1-phosphate, and α-d-xylose 1-phosphate (Sigma-Aldrich). The reactions contained 120 µm Teth514_1788 or 22 µm Teth514_1789 and were performed for 2 h at 30°C. The reaction products were analyzed using TLC as described in the preceding subsection.

### Structure Determination

The reaction products used for the structural studies were generated in 500- µL reaction mixtures (pH 5.0 for Teth514_1788; pH 5.5 for Teth514_1789) containing 50 mm α-Man1*P* plus 50 mm of each acceptor carbohydrate and Teth514_1788 (530 nm for d-mannose and d-fructose and 72 nm for β-1,2-mannobiose (β-1,2-Man_2_)) or Teth514_1789 (64 nm for d-mannose and d-fructose). The reaction mixtures were incubated at 30°C for 24 h and then desalted using Amberlite MB-4 (Organo, Tokyo, Japan). The reaction products were purified using an HPLC system (Prominence; Shimadzu, Kyoto, Japan) equipped with a Shodex Asahipak NH2P-50 4E column (4.6-mm internal diameter ×25 cm, 5 µm; Showa Denko K.K., Tokyo, Japan) at 30°C under a constant flow (1.0 ml·min^−1^) of 70% acetonitrile in water as the mobile phase. The fractions containing the reaction products were collected and lyophilized. The molecular masses of the products were determined using electrospray ionization MS (ESI-MS). The ESI-MS spectra were recorded in the positive-ion mode on a time-of-flight (TOF)-MS system (JMS-T100 LP AccuTOF LC-Plus; JEOL Co., Tokyo, Japan) equipped with an ESI source (JEOL Co.). The one-dimensional (^1^H and ^13^C) and two-dimensional (double-quantum filtered correlation spectroscopy (DQF-COSY), heteronuclear single-quantum coherence (HSQC), and heteronuclear multiple-bond correlation (HMBC)) NMR spectra of the products were acquired in D_2_O, using 2-methyl-2-propanol as an internal standard (*δ*_H_ 1.23 and *δ*_C_ 31.2), using a Bruker DMX 600 spectrometer (Bruker Biospin, Rheinstetten, Germany) or a Bruker Avance 800 spectrometer (Bruker Biospin). The proton signals were assigned based on the DQF-COSY spectra. The ^13^C signals were assigned using the HSQC spectra based on the assignment of the proton signals. The linkage position in each product was determined by detecting the inter-ring cross-peaks in each HMBC spectrum. The anomeric configuration was confirmed based on the ^1^ *J*_C1–H1_ coupling constant that was extracted from the non-decoupled HSQC spectrum.

### Measurement of Phosphorolytic Activity

The phosphorolysis substrates of Teth514_1788 and Teth514_1789 were generated in a 4-mL reaction mixture (pH 5.0) containing 14 µm Teth514_1788, 500 mm α-Man1*P*, and 500 mm d-mannose. After incubation at 30°C for 72 h, the reaction mixture was desalted using Amberlite MB-4. The reaction products were purified using an HPLC system equipped with a Shodex Asahipak NH2P-50 10E column (10-mm internal diameter ×25 cm; Showa Denko K.K.) at 30°C under a constant flow (3.0 ml·min^−1^) of 65% acetonitrile in water (the mobile phase). The fractions containing the products were collected and lyophilized. The phosphorolytic activity was routinely determined by quantifying the α-Man1*P* released during a phosphorolytic reaction in 40 mm MES-NaOH buffer (pH 6.0) containing 10 mm substrate and 10 mm P_i_ at 30°C using the previously described colorimetric method [25].

### Temperature and pH Profile

The effects of pH on the phosphorolytic and synthetic activities of Teth514_1788 (44 nm) and Teth514_1789 (32 nm) were measured under the standard conditions described above and the following 40 mm buffers were used: sodium citrate (pH 3.0–5.5), bis(2-hydroxyethyl)iminotris(hydroxymethyl)methane-HCl (pH 5.5–7.0), HEPES-NaOH (pH 7.0–8.5), and glycine-NaOH (pH 8.5–10.5). The thermal and pH stabilities were evaluated by measuring the residual synthetic activities (under the standard conditions) after incubating Teth514_1788 (360 nm) and Teth514_1789 (320 nm) at 30–90°C for 30 min or under various pH conditions at 4°C for 24 h, respectively.

### Kinetic Analysis

The initial velocities of the phosphorolytic reactions performed using 1,2-β-oligomannan were determined at 30°C under the standard conditions described above. We used Teth514_1788 (180 nm for β-1,2-Man_2_ and 36 nm for β-1,2-Man_3_ and β-1,2-Man_4_) and Teth514_1789 (16 nm for β-1,2-Man_2_, 32 nm for β-1,2-Man_3_ and β-1,2-Man_4_) with a combination of initial substrate concentrations (0.25, 0.50, 1.0, 2.0, 3.0, 5.0, and 10 mm) and P_i_ concentrations (0.10, 0.20, 0.30, 0.50, 1.0, and 2.0 mm). The kinetic parameters were calculated by curve-fitting the experimental data to the theoretical equation used for describing a sequential Bi Bi mechanism, *v*  =  *k*_cat_ [E]_0_[A][B]/(*K*_iA_K_mB_ + *K*_mB_ [A] + *K*_mA_ [B] + [A][B]) (A =  substrate, B =  P_i_), using GraFit Version 7.0.3 (Erithacus Software Ltd., London, UK).

We analyzed the kinetics of the synthetic reactions using suitable acceptors and the standard conditions described above. We used Teth514_1788 (290 nm for d-mannose and d-fructose and 29 nm for β-1,2-Man_2_ and β-1,2-Man_3_) or Teth514_1789 (28 nm for d-mannose, d-fructose, β-1,2-Man_2_, and 280 nm for β-1,2-Man_3_) with various acceptor concentrations (0.1, 0.2, 0.25, 0.3, 0.5, 1.0, 2.0, 3.0, 5.0, and 10 mm in the case of d-fructose used as the acceptor for Teth514_1788 or 0.25, 0.3, 0.5, 1.0, 2.0, 3.0, 5.0, and 10 mm for the other acceptors) and α-Man1*P* (0.5, 1.0, 2.0, 3.0, 5.0, and 10 mm) as the donor, together with 10 mm of each opposite substrate. The kinetic parameters were calculated by curve-fitting the experimental data to the Michaelis-Menten equation, *v*  =  *k*_cat_ [E]_0_ [S]/(*K*_m_ + [S]), using GraFit Version 7.0.3.

## Results

### Prediction of the Enzymatic Function of Teth514_1788 and Teth514_1789

**Figure 1:**
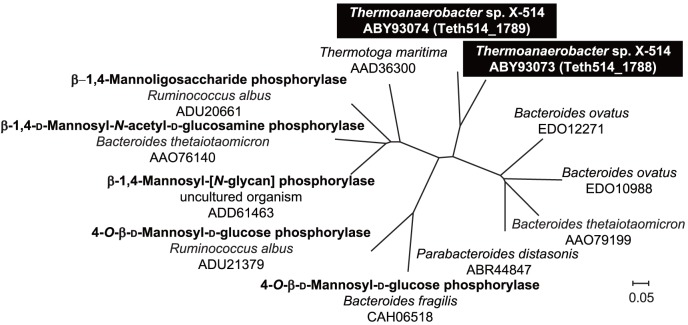
The phylogenetic tree of the GH130 proteins. The multiple alignments were performed using ClustalW2 (http://www.ebi.ac.uk/Tools/msa/clustalw2/). A phylogenetic tree was constructed using TreeView Version 1.6.6 (http://taxonomy.zoology.gla.ac.uk/rod/rod.html). The genes encoding the GH130 proteins (http://www.cazy.org/GH130.html) are represented according to the organism names and GenBank accession numbers. The genes cloned in this study are shown in white letters on a black background. The enzyme names of the characterized phosphorylases are shown in bold.

We noticed that the amino acid sequences of Teth514_1788 and Teth514_1789, which are encoded in the *Thermoanaerobacter* sp. X-514 genome, exhibit low sequence identities (20%–28%) with other GH130 β-d-mannoside phosphorylases, which include the 4-*O*-β-d-mannosyl-d-glucose phosphorylases from *B*. *fragilis* [13] and *R*. *albus* [14], a β-1,4-mannooligosaccharide phosphorylase from *R*. *albus* [14], a β-1,4-d-mannosyl-*N*-acetyl-d-glucosamine phosphorylase from *B*. *thetaiotaomicron* [10], and a β-mannopyranosyl-[*N*-glycan] phosphorylase from an uncultured human gut bacterium [16]. Furthermore, based on a phylogenetic tree analysis (Fig. 1), Teth514_1788 and Teth514_1789 could not be categorized into any of the characterized GH130 phosphorylases [10], [13], [14], [16]. The amino acid sequences of Teth514_1788 and Teth514_1789 showed no predicted N-terminal signal peptides based on a PSORTb Version 3.0.2 [18] and a SignalP 4.1 analysis [19]. These results suggest that Teth514_1788 and Teth514_1789 play a role in the intercellular phosphorolysis of certain β-mannoside. In this study, we expressed recombinant Teth514_1788 and Teth514_1789 in *E. coli* Rosetta 2 (DE3) to investigate their enzymatic properties and physiological roles in the bacterium.

### Preparation of the Recombinant Teth514_1788 and Teth514_1789

Recombinant Teth514_1788 and Teth514_1789 fused with a His_6_-tag at the C-terminus were purified using nickel-chelate affinity chromatography, with yields of 18 and 3.0 mg, respectively, from lysates prepared using 400 mL of cell culture. The purified Teth514_1788 and Teth514_1789 migrated in the SDS-PAGE as single protein bands with an estimated size of 34 kDa, which agrees with the theoretical molecular masses of 33,997 and 36,175, respectively. Furthermore, based on the gel filtration chromatography, the molecular masses of Teth514_1788 and Teth514_1789 were estimated to be 50 and 27 kDa, respectively. These results indicate that Teth514_1788 and Teth514_1789 exist as a homodimer and monomer in solution, respectively. In contrast, *B*. *thetaiotaomicron* GH130 β-1,4-d-mannosyl-*N*-acetyl-d-glucosamine phosphorylase [10] and *R*. *albus* GH130 β-1,4-mannooligosaccharide phosphorylase [14] have been reported to exist as a homotetramer and homohexamer, respectively.

### Acceptor and Donor Specificities of Teth514_1788 and Teth514_1789 in Synthetic Reactions

**Figure 2:**
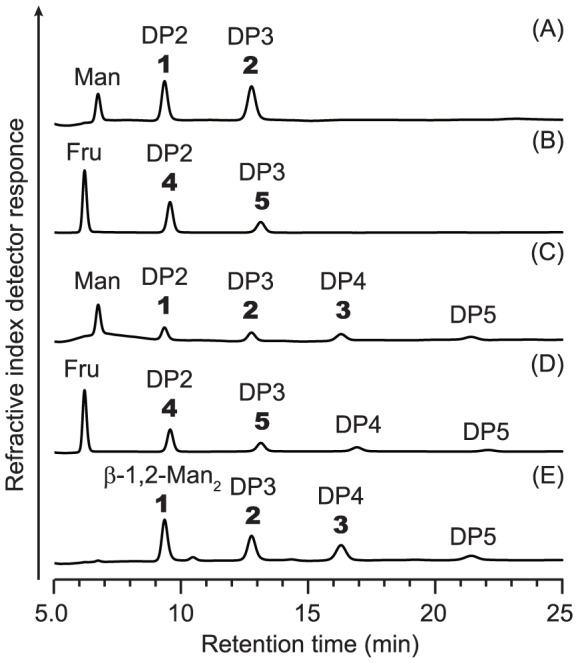
The HPLC profiles of the products of the synthetic reactions performed using suitable carbohydrate acceptors with α-Man1*P* as the donor. The reaction mixtures contained (*A*) 50 mm d-mannose, 50 mm α-Man1*P*, and 64 nm Teth514_1789; (*B*) 50 mm d-fructose, 50 mm α-Man1*P*, and 64 nm Teth514_1789; (*C*) 50 mm d-mannose, 50 mm α-Man1*P*, and 530 nm Teth514_1788; (*D*) 50 mm d-fructose, 50 mm α-Man1*P*, and 530 nm Teth514_1788; and (*E*) 50 mm β-1,2-Man_2_, 50 mm α-Man1*P*, and 72 nm Teth514_1788. The mixtures were incubated at 30°C for 24 h and then desalted using Amberlite MB-4. The reaction products were analyzed using an HPLC system equipped with a Shodex Asahipak NH2P-50 4E column (4.6-mm internal diameter ×25 cm, 5 µm) at 30°C under a constant flow (1.0 ml·min^−1^) of 70% acetonitrile in water (mobile phase). The product numbers described in the text are in bold. The fractions containing the reaction products were collected and lyophilized. The amounts of products 1 and 2 obtained from d-mannose (*A* and *C*) were 1.5 and 1.2 mg (Teth514_1789) and 0.8 and 1.0 mg (Teth514_1788), respectively. The amounts of products 4 and 5 obtained from D-fructose (*B* and *D*) were 1.4 and 2.3 mg (Teth514_1789) and 1.0 and 0.5 mg (Teth514_1788), respectively. The amounts of products 2 and 3 obtained from β-1,2-Man_2_ (*E*) using Teth514_1788 were 1.0, and 1.3 mg, respectively. The masses were as follows: products 1, 2, 3, 4, and 5 were m/z 365.1, 527.2, 689.2, 365.1, and 527.2 [M+Na]^+^, respectively.

**Figure 3:**
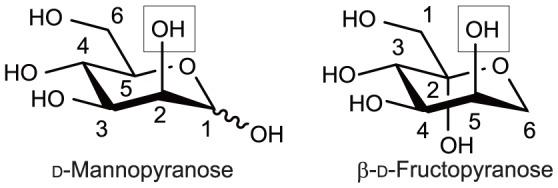
A comparison of the orientations of the substituting groups between d-mannopyranose and d-fructopyranose. Pyranose rings are depicted to align with the linkage positions that are shown in the box.

The acceptor specificity in a synthetic reaction was examined using various carbohydrate acceptor candidates (see “Materials and Methods”) together with α-Man1*P* as the donor. Teth514_1789 used d-mannose and d-fructose as the suitable acceptors. The synthetic reaction performed using d-mannose generated products **1** and **2** (Fig. 2A), and their structures were determined using ^1^H and ^13^C NMR spectroscopy. The results of the HMBC experiments showed that each product exhibited correlation cross-peaks between the C2 of the d-mannose residues on the acceptor side and the H-1 of the d-mannosyl residues on the donor side. The ^1^ *J*_C1–H1_ coupling constant (160∼162 Hz) of the d-mannosyl residues on the donor side were consistent with β-linkages [26]. The assignments of the ^1^H and ^13^C NMR chemical shifts are provided in S1 Table. Based on these results, we identified products **1** and **2** as β-d-mannopyranosyl-(1→2)-d-mannose (β-1,2-Man_2_) and β-d-mannopyranosyl-(1→2)-β-d-mannopyranosyl-(1→2)-d-mannose (β-1,2-Man_3_), respectively. The synthetic reaction performed using d-fructose also generated two products (products **4** and **5**, Fig. 2B). In the ^1^H and ^13^C NMR spectrographs of products **4** and **5**, the liner form of d-fructose and α-d-fructopyranose were not detected. Based on the structural analyses, we identified products **4** and **5** as β-d-mannopyranosyl-(1→5)-β-d-fructopyranose and β-d-mannopyranosyl-(1→2)-β-d-mannopyranosyl-(1→5)-β-d-fructopyranose, respectively (the chemical shifts are summarized in S2 Table). The acceptor preference can be explained by the ring conformations and the orientations of the substituting groups of d-mannose and d-fructose. When the hydroxyl groups at the linkage positions of d-mannopyranose and β-d-fructopyranose are aligned, the orientations of the hydroxyl groups at C2, C3, C4, and C6 of d-mannopyranose and at C5, C4, C3, and C1 of β-d-fructopyranose are conserved (Fig. 3). Therefore, the β-1,5-linkage on β-d-fructopyranose and the β-1,2-linkage on d-mannopyranose will be formed. Although d-mannose and d-fructose also acted as acceptors with Teth514_1788, the formation of oligosaccharides containing a degree of polymerization (DP) between 2–5 were detected (Fig. 2C and 3D). The production of oligosaccharides with a DP ≥4 by Teth514_1789 was not detected under the reaction conditions (Fig. 2A and 2B). The ^1^H NMR spectra of the oligosaccharides that were generated from d-mannose and d-fructose and containing a DP between 2–3 were identical to the spectra of products **1** and **2** from d-mannose and products **4** and **5** from d-fructose with Teth514_1789 (S1 Figure). Furthermore, we confirmed that Teth514_1788 used product **1** (β-1,2-Man_2_) (Fig. 3E) as the acceptor, and this resulted in the synthesis of oligosaccharides with DP 3 (product **2**), DP4 (product **3**), and DP5. Structurally, products **2** and **3** were identified to be β-1,2-Man_3_ and β-d-mannopyranosyl-(1→2)-β-d-mannopyranosyl-(1→2)-β-d-mannopyranosyl-(1→2)-d-mannose (β-1,2-Man_4_) (S2 Figure). These results indicate that Teth514_1788 and Teth514_1789 successively transfer a β-mannopyranosyl residue from α-Man1*P* to the synthesized 1,2-β-oligomannosyl chain, and Teth514_1788 can produce 1,2-β-oligomannan containing a greater DP than those produced by Teth514_1789. The donor specificity in the synthetic reaction was also examined using various sugar-phosphate derivatives (see “Materials and Methods”). Teth514_1788 and Teth514_1789 exhibited no activity toward any of the other sugar-phosphate derivatives that were examined. These results agree with the finding that the amino acid residues involved in the recognition of d-mannose at subsite -1 in 4-*O*-β-d-mannosyl-d-glucose phosphorylase from *B*. *fragilis* (Asn73, Asp131, and Asp344), the mechanism of which was identified based on three-dimensional structural analyses [27], are conserved in Teth514_1788 (Asn28, Asp92, and Asp281) and Teth514_1789 (Asn28, Asp98, and Asp287) (S3 Figure).

### Kinetic Analysis of the Synthetic and Phosphorolytic Reactions

We determined the kinetic parameters of the four acceptors (d-mannose, d-fructose, β-1,2-Man_2_, and β-1,2-Man_3_) used by Teth514_1788 and Teth514_1789 to investigate the acceptor preference in the presence of the α-Man1*P* donor (Table 1). The *k*_cat_/*K*_m_ value of d-mannose for Teth514_1789 was highest among the four tested acceptors. The *k*_cat_/*K*_m_ value of β-1,2-Man_2_ was 6 times lower than that of d-mannose, and no obvious activity was observed against β-1,2-Man_3_. These results indicate that d-mannose is the most effective acceptor for Teth514_1789. In regards to Teth514_1788, the *k*_cat_/*K*_m_ values of β-1,2-Man_2_ and β-1,2-Man_3_ were 7–8 times greater than that of d-mannose. These kinetic parameters indicate that Teth514_1788 and Teth514_1789 exhibit distinct chain-length specificities toward 1,2-β-oligomannan in synthetic reactions. The kinetic parameters measured for α-Man1*P* are similar to other inverting phosphorylases for their specific donors [3], [10], [13], [14], [16], [28]–[30].

**Table 1:** The kinetic parameters for the synthetic reactions catalyzed by Teth514_1788 and Teth514_1789.

	*k*_cat_ (s^−1^)	*K*_m_ (mm)	*k*_cat/_ *K*_m_ (s^−1^mm ^−1^)
**Teth514_1788**			
Acceptor^a^			
d-Mannose	1.9±0.1	0.41±0.07	4.6
d-Fructose	2.0±0.1	0.20±0.03	10
β-1,2-Man_2_	25±1	0.77±0.2	32
β-1,2-Man_3_	43±2	1.2±0.2	36
Donor*^b^*			
α-Man1*P*	22±2	1.4±0.3	16
**Teth514_1789**			
Acceptor^a^			
d-Mannose	10±0.2	0.46±0.05	22
d-Fructose	11±1	0.69±0.1	16
β-1,2-Man_2_	7.0±0.3	1.8±0.3	3.9
β-1,2-Man_3_	–d	–d	–d
Donor*^c^*			
α-Man1*P*	12±2	2.1±0.8	5.7

a The kinetic parameters were calculated by fitting the initial velocities to various concentrations of acceptor substrates in the presence of 10 mm α-Man1*P* using the Michaelis-Menten equation.

b,c The kinetic parameters were calculated by fitting the initial velocities toward various concentrations of donor substrates in the presence of 10 mm β-1,2-Man_2_ (*b*) or 10 mm d-mannose (*c*) using the Michaelis-Menten equation.

d Not detectable. To investigate the acceptor specificities, the synthetic reactions were examined using the following putative carbohydrate acceptors: d-allose, d-altrose, d-arabinose, d-fructose, d-galactosamine, d-galactose, d-galacturonic acid, d-glucosamine, d-glucose, d-glucuronic acid, d-lyxose, d-talose, d-ribose, d-xylose, l-arabinose, l-rhamnose, 1,5-anhydro-d-glucitol, 2-deoxy-d-glucose, methyl-α-d-glucoside, methyl-β-d-glucoside, 3-*O*-methyl-d-glucose, *N*-acetyl-d-galactosamine, *N*-acetyl-d-glucosamine, *N*-acetyl-d-mannosamine, cellobiose, gentiobiose, isomaltose, kojibiose, lactose, lactulose, laminaribiose, maltose, melibiose, nigerose, *N*,*N*′-diacetylchitobiose, sophorose, sucrose, trehalose, and xylobiose.

**Figure 4:**
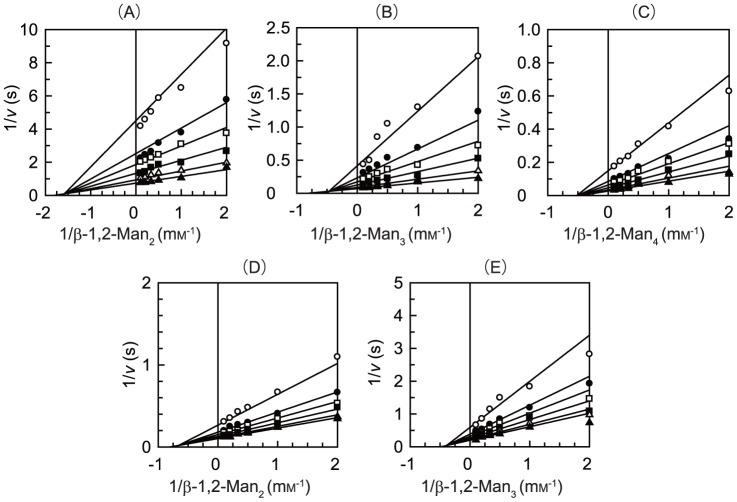
Double-reciprocal plots of the phosphorolysis catalyzed by Teth514_1788 (*A*, *B*, and *C*) and Teth514_1789 (*D* and *E*). We measured the initial velocities for the phosphorolysis of 1,2-β-oligomannan at various concentrations of 1,2-β-oligomannan and P_i_. Open circle, 0.1 mm P_i_; closed circle, 0.2 mm P_i_; open square, 0.3 mm P_i_; closed square, 0.5 mm P_i_; open triangle, 1 mm P_i_; and closed triangle, 2 mm P_i_.

Teth514_1788 and Teth514_1789 phosphorolyzed 1,2-β-oligomannan with an inversion of the anomeric configuration to release α-Man1*P*. Moreover, Teth514_1788 and Teth514_1789 did not cleave the 1,2-β-oligomannan in the absence of P_i_. Double reciprocal plots of the initial velocities against various initial concentrations of 1,2-β-oligomannan and P_i_ gave a series of lines that intersected at a single point (Fig. 4). These results indicate that the phosphorolytic reactions follow a sequential Bi Bi mechanism, which has also been reported for other inverting phosphorylases [3], [10], [13], [14], [16], [28]–[32]. Teth514_1788 and Teth514_1789 did not phosphorolyze β-1,4-d-mannosyl-d-glucose, β-1,4-mannooligosaccharides (DP 2–3), and β-1,4-d-mannosyl-*N*-acetyl-d-glucosamine, which are known substrates for the GH130 4-*O*-β-d-mannosyl-d-glucose phosphorylase [13], [14], β-1,4-mannooligosaccharide phosphorylase [14], and β-1,4-d-mannosyl-*N*-acetyl-d-glucosamine phosphorylase [10], respectively. The kinetic parameters for the phosphorolytic reactions on 1,2-β-oligomannan are summarized in Table 2. β-1,2-Man_2_ (31 mg), β-1,2-Man_3_ (24 mg), and β-1,2-Man_4_ (21 mg) prepared for the kinetics analysis were used as the substrates (see “Materials and Methods”). The *k*_cat_/*K*_m_ value of β-1,2-Man_2_ for Teth514_1789 was 3 times higher than that of β-1,2-Man_3_, and the phosphorolytic activity toward β-1,2-Man_4_ was not detectable. These results indicate that β-1,2-Man_2_ is the best substrate for the phosphorolytic reaction of Teth514_1789. In regards to Teth514_1788, the *k*_cat_/*K*_m_ values of β-1,2-Man_3_ and β-1,2-Man_4_ were 6 times greater than that of β-1,2-Man_2_. These kinetic parameters indicate that Teth514_1788 prefers 1,2-β-oligomannan with a DP ≥3 and Teth514_1789 prefers β-1,2-Man_2_and that the two enzymes exhibit distinct chain-length specificities toward 1,2-β-oligomannan in phosphorolytic reactions. The kinetic parameters measured for 1,2-β-oligomannan are similar to those measured using other inverting phosphorylases [3], [10], [13], [14], [16], [29], [31], [32], which indicates that 1,2-β-oligomannans containing β-1,2-Man_2_ are the true substrates of Teth514_1788 and Teth514_1789.

**Table 2:** The kinetic parameters for the phosphorolytic reactions catalyzed by Teth514_1788 and Teth514_1789.

	*k*_cat_ (s^−1^)	*K*_mA_ (mm)	*K*_mB_ (mm)	*K*_ia_ (mm)	*k*_cat/_ *K*_mA_ (s^−1^mm ^−1^)
**Teth514_1788**					
β-1,2-Man_2_	1.9±0.1	0.52±0.1	0.74±0.1	0.63±0.2	3.7
β-1,2-Man_3_	20±0.2	0.92±0.2	0.71±0.1	2.1±0.5	22
β-1,2-Man_4_	49±4	2.3±0.4	0.63±0.1	1.9±0.6	21
**Teth514_1789**					
β-1,2-Man_2_	11±0.3	1.2±0.1	0.18±0.02	1.5±0.4	8.8
β-1,2-Man_3_	5.6±0.4	2.0±0.4	0.23±0.05	2.6±1	2.8
β-1,2-Man_4_	–*^a^*	–*^a^*	–*^a^*	–*^a^*	–*^a^*

The kinetic parameters were calculated by fitting the initial velocities toward 0.25–10 mm substrate in the presence of 0.1–2.0 mm P_i_ using the following theoretical equation for a sequential Bi Bi mechanism (using GraFit Version 7.0.3): *v*  =  *k*_cat_[E]_0_[A][B]/(*K*_iA_ *K*_mA_ + *K*_mA_[B] + *K*_mB_[A] + [A][B]), where A is the substrate and B is P_i_. *^a^* Not detectable.

Based on the above information, we propose 1,2-β-oligomannan:phosphate α-d-mannosyltransferase as the systematic name and 1,2-β-oligomannan phosphorylase as the short name for Teth514_1788 and β-1,2-mannobiose:phosphate α-d-mannosyltransferase as the systematic name and β-1,2-mannobiose phosphorylase as the short name for Teth514_1789.

### Basic Properties of Teth514_1788 and Teth514_1789

**Figure 5:**
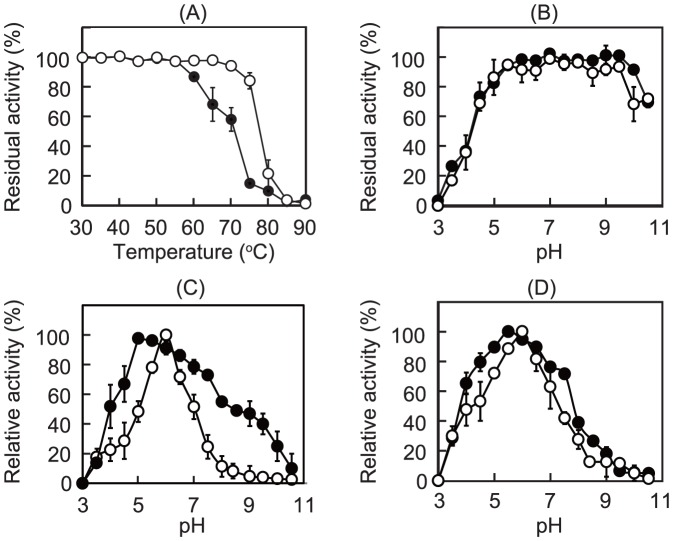
The effects of pH and temperature on the activities and stabilities of Teth514_1788 and Teth514_1789. (*A*) The thermal stabilities of 360 nm Teth514_1788 (closed symbols) and 320 nm Teth514_1789 (open symbols) at a temperature range between 30–90°C for 30 min. (*B*) The pH stabilities of 360 nm Teth514_1788 (closed symbols) and 320 nm Teth514_1789 (open symbols) at 4°C for 24 h. (*C*) The pH dependence on the phosphorolytic and synthetic activities of Teth514_1788 (44 nm) in 40 mm sodium citrate (pH 3.0–5.5), bis(2-hydroxyethyl)iminotris(hydroxymethyl)methane-HCl (pH 5.5–7.0), HEPES-NaOH (pH 7.0–8.5), and glycine-NaOH (pH 8.5–10.5). (*D*) The pH dependence of the phosphorolytic and synthetic activities of Teth514_1789 (32 nm) in the same buffers listed in Panel C. In Panels C and D, the closed and open symbols represent the synthetic and phosphorolytic activities, respectively.

Teth514_1788 and Teth514_1789 were stable up to 55 and 75°C, respectively, during a 30 min incubation (Fig. 5A) and were stable in pH ranges between 4.0–9.5 and 5.5–9.5, respectively, at 4°C for 24 h (Fig. 5B). Furthermore, both Teth514_1788 and Teth514_1789 exhibited the highest apparent phosphorolytic activity at pH 6.0 (Fig. 5C and 5D), whereas the optimal pH values for the synthetic reactions were 5.0 and 5.5, respectively (Fig. 5C and 5D).

## Discussion

### The Enzyme Functions of Teth514_1788 and Teth514_1789

In this study, we identified Teth514_1788 and Teth514_1789 as new members of the GH130 phosphorylase, which catalyze the reversible phosphorolysis of 1,2-β-oligomannan. The two unique phosphorylases, Teth514_1788 and Teth514_1789, can be distinguished by comparing their chain-length specificities toward 1,2-β-oligomannan. In phosphorolytic reactions, Teth514_1789 prefers β-1,2-Man_2_, whereas Teth514_1788 shows substrate specificity toward 1,2-β-oligomannan with a DP ≥3, similar to the chain-length specificities observed in the GH65 α-1,3-oligoglucan phosphorylase [29], the GH94 cellodextrin phosphorylase [33], the GH94 1,2-β-oligoglucan phosphorylase [34], and the GH130 β-1,4-mannooligosaccharide phosphorylase [14].

A recent phylogenetic tree analysis classified the GH130 enzymes into three subfamilies (S3 Figure) [16]. The subfamily GH130_1 includes 4-*O*-β-d-mannosyl-d-glucose phosphorylases from *B*. *fragilis* (BF0772) [13] and *R*. *albus* (Rumal_0852) [14], which exhibit a narrow substrate specificity toward 4-*O*-β-d-mannosyl-d-glucose. The subfamily GH130_2 includes β-1,4-mannooligosaccharide phosphorylase from *R*. *albus* (Rumal_0099) [14], β-1,4-d-mannosyl-*N*-acetyl-d-glucosamine phosphorylase from *B*. *thetaiotaomicron* (BT_1033) [10], and β-mannopyranosyl-[*N*-glycan] phosphorylase from an uncultured human gut bacterium (UhgbMP) [16], in addition to TM1225 from *Thermotoga maritima* (PDB accession code 1VKD). The GH130_NC cluster contains four proteins with known structures: BDI_3141 from *Parabacteroides distasonis* (PDB accession code 3TAW), BT_4094 from *B. thetaiotaomicron* (3R67), and BACOVA_03624 and BACOVA_02161 from *B. ovatus* (3QC2 and 4ONZ). Teth514_1788 and Teth514_1789, which were the focus of this study, are also part of the GH130_NC cluster. A key residue position occupied by Tyr103 in UhgbMP (Tyr130 in BF0772) allows the GH130_NC sequences to be discriminated from those of the other subfamilies (S3 Figure) [16]. The Tyr residue is strictly conserved in the GH130_1 and GH130_2 subfamilies, but it is replaced by a Glu residue in GH130_NC. Moreover, the majority of the enzymes belonging to the GH130_NC cluster are suspected to be hydrolases—and not phosphorylases—in which the Glu residue acts as the catalytic general base [16]. However, in this study, our results clearly demonstrated that both Teth514_1788 and Teth514_1789, which possess the Glu residue (Glu91 and Glu97, respectively) that is conserved in the GH130_NC cluster, catalyzed the reversible phosphorolysis of 1,2-β-oligomannan.

The two phosphorylases described herein are the first to be reported to degrade 1,2-β-mannosidic linkages. 1,2-β-oligomannan contains a unique structure that has been reported only in microorganisms [35]–[43], and it was first identified as phosphopeptidomannan in *Candida albicans* [35]. The *C. albicans* epitopes that consist of 1,2-β-oligomannan are involved in the adhesion of *C. albicans* to the macrophage membrane [44], [45], which can elicit the generation of protective antibodies [46] and induce cytokine production [47]. Furthermore, the structure is also found in the lipopolysaccharide *O*-antigens of *Klebsiella pneumoniae* [36], *Salmonella* species [37], *Burkholderia cepacia* [40], and *E*. *coli* [41], [42], and it is present in the intracellular and extracellular polysaccharides of the trypanosoma protozoa *Crithidia* and *Herpetomonas* [38], and in *Leishmania mexicana* [39], [43]. In *L. mexicana*, β-1,2-mannan (DP of 4–40) functions as a carbohydrate reserve for the parasite's survival in host macrophages and constitutes 80%–90% of the cellular carbohydrates during the developmental stages [39]. However, no potential source of 1,2-β-oligomannan has been identified in the genus *Thermoanaerobacter*. Therefore, Teth514_1788 and Teth514_1789 may be involved in salvaging these polysaccharides.

### The Physiological Role of GH130 Phosphorylases in *Thermoanaerobacter*

**Figure 6:**
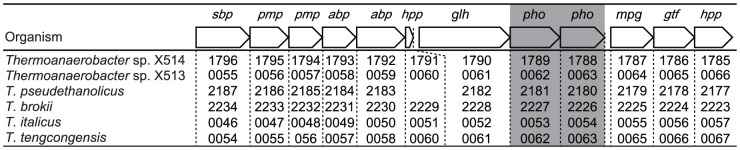
The gene loci in the gene cluster involved in GDP-d-mannose biosynthesis in the genus *Thermoanaerobacter*. Gray rectangle, the GH130 β-1,2-mannoside phosphorylase (*pho*) of focus in this study; *abp*, ABC transporter ATP-binding protein; *sbp*, extracellular solute-binding protein; *glh*, GH5 β-glycoside hydrolase; *gtf*, GT4 glycosyltransferase; *hpp*, hypothetical protein; *mpg*, mannose-1-phosphate guanylyltransferase; and *pmp*, ABC transporter permease protein.

**Figure 7:**
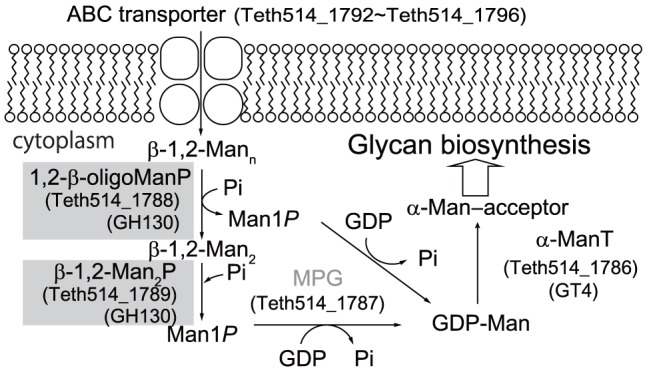
The proposed metabolic pathway, including GDP-d-mannose biosynthesis, in *Thermoanaerobacter* sp. X-514. The genes cloned in this study are shown on a gray background. GDP-Man, GDP-d-mannose; MPG, mannose-1-phosphate guanylyltransferase; α-ManT, GDP-mannose-dependent α-mannosyltransferase; 1,2-β-oligoManP, 1,2-β-oligomannan phosphorylase; β-1,2-Man_2_P, β-1,2-mannobiose phosphorylase; and PP_i_, diphosphate.

We found that the two genes encoding Teth514_1788 and Teth514_1789 were located in a gene cluster that was predicted to be involved in GDP-d-mannose biosynthesis (Fig. 6). The gene cluster is suggested to be highly conserved in the genomes of the members of the genus *Thermoanaerobacter* (Fig. 6), including *Thermoanaerobacter* sp. X-514 (GenBank accession number CP000923) [20], *Thermoanaerobacter* sp. X-513 (CP002210) [20], *T. brockii* subsp. *finnii* ATCC 43586 (CP002466) [20], *T. pseudethanolicus* ATCC 33223 (CP000924) [20], *T. italicus* Ab9 (CP001936) [20], and *T. tengcongensis* MB4 (AE008691) [48]. The BLASTP assignment [17] (S3 Table) revealed that the gene cluster contained 12 unidirectionally transcribed ORFs (Fig. 1) that are predicted to encode five ATP-binding cassette (ABC) transporter proteins (Teth514_1796, which is an extracellular solute-binding protein; Teth514_1795 and Teth514_1794, which are permease proteins; and Teth514_1793 and Teth514_1792, which are ATP-binding proteins), two hypothetical proteins (Teth514_1791 and Teth514_1785), a GH5 β-glycoside hydrolase (Teth514_1790), a mannose-1-phosphate guanylyltransferase (Teth514_1787), a GDP-mannose-dependent α-mannosyltransferase (Teth514_1786), and two GH130 β-1,2-mannoside phosphorylases (Teth514_1788 and Teth514_1789). These sequence analyses indicated that the two phosphorylases may play a role in the intercellular phosphorolysis of β-1,2-mannoside to supply α-Man1*P* for GDP-d-mannose biosynthesis. However, to date, no reports have described a GDP-d-mannose biosynthesis pathway in which phosphorylases participate. We here propose a novel biosynthetic pathway for GDP-d-mannose production in *Thermoanaerobacter* sp. X-514 based on the experimental results of characterization of the two GH130 phosphorylase (Teth514_1788 and Teth514_1789) and the function prediction by BLASTP assignment as described above (S3 Table). GDP-d-mannose has typically been considered to be synthesized through the following three conventional pathways via Man6*P*: i) a *de novo* pathway that involves the successive conversion of d-glucose into Man6*P* via d-glucose 6-phosphate and d-fructose 6-phosphate by the sequential reactions of ATP-dependent carbohydrate kinase (Teth514_0049), glucose-6-phosphate isomerase (Teth514_1208), and mannose-6-phosphate isomerase (Teth514_1319) [49], [50]; ii) a salvage pathway in which d-mannose is directly converted into Man6*P* by ATP-dependent carbohydrate kinase (Teth514_0049) [49], [50]; or iii) a phosphoenolpyruvate-dependent carbohydrate phosphotransferase system (containing mannose-specific IIA (Teth514_0132), IIC (Teth514_0133), IID (Teth514_0134), and IIB (Teth514_0135) components) that is a concomitant carbohydrate-uptake and phosphorylation pathway in bacteria [51]. The resultant Man6*P* is commonly converted into GDP-d-mannose via α-Man1*P* by the sequential reactions of phosphomannomutase (Teth514_2238) and mannose-1-phosphate guanylyltransferase (Teth514_2275). In contrast to these pathways, we propose a different pathway for GDP-d-mannose biosynthesis in *Thermoanaerobacter* sp. X-514 (Fig. 7): 1,2-β-oligomannan is transported into the cytoplasmic space by an ABC transporter (Teth514_1792 to Teth514_1796) and is sequentially phosphorolyzed by 1,2-β-oligomannan phosphorylases (Teth514_1788) and β-1,2-mannobiose phosphorylases (Teth514_1789). Then, the resultant α-Man1*P* is converted into GDP-d-mannose by mannose-1-phosphate guanylyltransferase (Teth514_1787). In this pathway, Teth514_1788 and Teth514_1789, which exhibit distinct chain-length specificities, play a key role in the efficient supply of α-Man1*P*, which is the precursor of GDP-d-mannose. This is the first report of a salvage pathway for GDP-d-mannose biosynthesis in which phosphorylases participate. One notable feature of this new GDP-d-mannose biosynthetic pathway is that it allows anaerobes such as *Thermoanaerobacter* to use the energy from ATP more efficiently than via conventional *de novo* and salvage pathways where ATP-dependent hexokinase participates; this is because d-mannose can be phosphorylated without the consumption of ATP. We further suggest that this salvage pathway for GDP-d-mannose biosynthesis is a common pathway in *Thermoanaerobacter* (Fig. 6).

### Conclusions

Two novel phosphorylases, Teth514_1788 and Teth514_1789, belonging to glycoside hydrolase family 130, were characterized from *Thermoanaerobacter* sp. X-514. Teth514_1788 and Teth514_1789 catalyzed the reversible phosphorolysis of 1,2-β-oligomannan in a typical sequential Bi Bi mechanism using 1,2-β-oligomannan containing a DP ≥3 or β-1,2-Man_2_ as the optimal substrates, respectively. These results indicate that the two phosphorylases exhibit distinct chain-length specificities towards 1,2-β-oligomannan. Here, we propose Teth514_1788 and Teth514_1789 to be the novel 1,2-β-oligomannan phosphorylase and β-1,2-mannobiose phosphorylase, respectively.

## Supporting Information

S1 Figure A comparison of the ^1^H NMR spectra of the products from the synthetic reactions catalyzed by Teth514_1788 and Teth514_1789 from d-mannose and d-fructose. The spectra were taken in D_2_O, using 2-methyl-2-propanol as an internal standard (*δ*_H_ 1.23 and *δ*_C_ 31.2), using a Bruker DMX 600 spectrometer. Spectra (*A*) and (*C*) correspond to products **1** and **2**, respectively, and were obtained via the synthetic reaction of Teth514_1789 from d-mannose. Spectra (*E*) and (*G*) correspond to products 4 and 5, respectively, and were obtained via the synthetic reaction of Teth514_1789 from d-fructose. Spectra (*B*) and (*D*) correspond to products **1** and **2**, respectively, and were obtained via the synthetic reaction of Teth514_1788 from d-mannose. Spectra (*F*) and (*H*) correspond to products **4** and **5**, respectively, and were obtained via the synthetic reaction of Teth514_1788 from d-fructose.
(PDF)

S2 Figure The NMR spectra of product 3 from the synthetic reaction catalyzed by Teth514_1788 with the substrates β-1,2-mannobiose and α-Man1*P*. Product **3** was identified to be β-d-mannopyranosyl-(1→2)-β-d-mannopyranosyl-(1→2)-β-d-mannopyranosyl-(1→2)-d-mannose (β-1,2-Man_4_). The spectra were taken in D_2_O, using 2-methyl-2-propanol as an internal standard (*δ*_H_ 1.23 and *δ*_C_ 31.2), using a Bruker DMX 800 spectrometer. The terms I, II, III, and IV on the spectra indicate reducing, two internal, and non-reducing d-mannose residues, respectively. The numbers after the letters indicate the positions on each sugar. (A) ^1^H NMR spectrum; (B) ^13^C NMR spectrum; (C) HSQC spectrum; and (D) HMBC spectrum.
(PDF)

S3 Figure A multiple alignment of the GH130 homologs. The multiple alignment was performed using ClustalW2 (http://www.ebi.ac.uk/Tools/msa/clustalw2/). BF0772, 4-*O*-β-d-mannosyl-d-glucose phosphorylase from *Bacteroides fragilis* (GenBank accession number CAH06518); Rumal_0852, 4-*O*-β-d-mannosyl-d-glucose phosphorylase from *Ruminococcus albus* (ADU21379); Rumal_0099, β-1,4-mannooligosaccharide phosphorylase from *R*. *albus* (ADU20661); BT_1033, β-1,4-d-mannosyl-*N*-acetyl-d-glucose phosphorylase from *B*. *thetaiotaomicron* (AAO76140); UhgbMP, β-mannopyranosyl-[*N*-glycan] phosphorylase from an uncultured human gut bacterium (ADD61463); TM1225, GH130 protein from *Thermotoga maritime (*AAD36300*)*; BT_4094, GH130 protein from *B. thetaiotaomicron* (AAO79199); BDI_3141, GH130 protein from *Parabacteroides distasonis* (ABR44847); BACOVA_03624 and BACOVA_021614, GH130 proteins from *B. ovatus* (EDO10988 and EDO12271, respectively); Teth514_1788, 1,2-β-oligomannan phosphorylase from *Thermoanaerobacter* sp. X-514 (ABY93073); and Teth514_1789, β-1,2-mannobiose phosphorylase from *Thermoanaerobacter* sp. X-514 (ABY93074). Above the sequences, the secondary structures of BF0772 are shown as squiggles (α-helices), arrows (β-strands), and 'T' characters (β-turns). The strictly conserved residues are shown in a box with white characters. Residues that have similarity across the sequences are shown in a box with bold characters, indicating the similar residues in the sequence group. Black starred characters represent amino acid residues that are involved in the mannose recognition site of BF0772 (Asn73, Asp131, and Asp344).
(PDF)

S1 Table The chemical shifts in the ^1^H and ^13^C NMR spectra of the products of the synthetic reaction catalyzed by Teth514_1789 with the substrates d-mannose and α-Man1*P*.
(PDF)

S2 Table The chemical shifts in the ^1^H and ^13^C NMR spectra of the products of the synthetic reaction catalyzed by Teth514_1789 with the substrates d-fructose and α-Man1*P*.
(PDF)

S3 Table The deduced amino acid sequence similarities of the genes involved in GDP-d-mannose biosynthesis in *Thermoanaerobacter* sp. X-514. The similarities between the amino acid sequences were investigated using the BLASTP program (Swiss-Prot/TrEMBL database).
(PDF)
