# Supplementary material for: Discovery of Two β-1,2-Mannoside Phosphorylases Showing Different Chain-Length Specificities from Thermoanaerobacter sp. X-514
Source: PLoS One. 2014 Dec 12;9(12):e114882. doi: 10.1371/journal.pone.0114882 (PMC4264767; doi:10.1371/journal.pone.0114882)
Supplement: S1 Figure — A comparison of the 1H NMR spectra of the products from the synthetic reactions catalyzed by Teth514_1788 and Teth514_1789 from d-mannose and d-fructose. The spectra were taken in D2O, using 2-methyl-2-propanol as an internal standard (δ H 1.23 and δ C 31.2), using a Bruker DMX 600 spectrometer. Spectra (A) and (C) correspond to products 1 and 2, respectively, and were obtained via the synthetic reaction of Teth514_1789 from d-mannose. Spectra (E) and (G) correspond to products 4 and 5, respectively, and were obtained via the synthetic reaction of Teth514_1789 from d-fructose. Spectra (B) and (D) correspond to products 1 and 2, respectively, and were obtained via the synthetic reaction of Teth514_1788 from d-mannose. Spectra (F) and (H) correspond to products 4 and 5, respectively, and were obtained via the synthetic reaction of Teth514_1788 from d-fructose. (PDF) [file pone.0114882.s001.pdf]

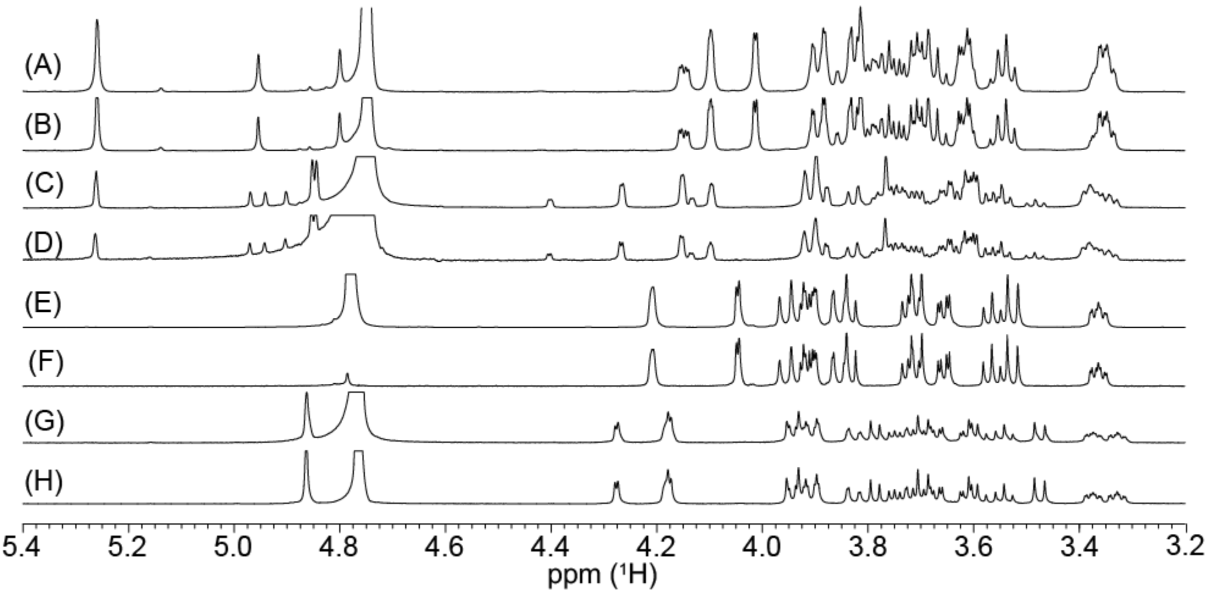

Figure S1. A comparison of the ^{1}H NMR spectra of the products from the synthetic reactions catalyzed by Teth514_1788 and Teth514_1789 from D-mannose and D-fructose.

The spectra were taken in D_2O, using 2-methyl-2-propanol as an internal standard (δ_H 1.23 and δ_C 31.2), using a Bruker DMX 600 spectrometer. Spectra (*A*) and (*C*) correspond to products **1** and **2**, respectively, and were obtained via the synthetic reaction of Teth514_1789 from D-mannose. Spectra (*E*) and (*G*) correspond to products 4 and 5, respectively, and were obtained via the synthetic reaction of Teth514_1789 from D-fructose. Spectra (*B*) and (*D*) correspond to products **1** and **2**, respectively, and were obtained via the synthetic reaction of Teth514_1788 from D-mannose. Spectra (*F*) and (*H*) correspond to products **4** and **5**, respectively, and were obtained via the synthetic reaction of Teth514_1788 from D-fructose.
