# Supplementary material for: Discovery of Two β-1,2-Mannoside Phosphorylases Showing Different Chain-Length Specificities from Thermoanaerobacter sp. X-514
Source: PLoS One. 2014 Dec 12;9(12):e114882. doi: 10.1371/journal.pone.0114882 (PMC4264767; doi:10.1371/journal.pone.0114882)
Supplement: S2 Figure — The NMR spectra of product 3 from the synthetic reaction catalyzed by Teth514_1788 with the substrates β-1,2-mannobiose and α-Man1P. Product 3 was identified to be β-d-mannopyranosyl-(1→2)-β-d-mannopyranosyl-(1→2)-β-d-mannopyranosyl-(1→2)-d-mannose (β-1,2-Man4). The spectra were taken in D2O, using 2-methyl-2-propanol as an internal standard (δ H 1.23 and δ C 31.2), using a Bruker DMX 800 spectrometer. The terms I, II, III, and IV on the spectra indicate reducing, two internal, and non-reducing d-mannose residues, respectively. The numbers after the letters indicate the positions on each sugar. (A) 1H NMR spectrum; (B) 13C NMR spectrum; (C) HSQC spectrum; and (D) HMBC spectrum. (PDF) [file pone.0114882.s002.pdf]

(A) HSQC spectrum

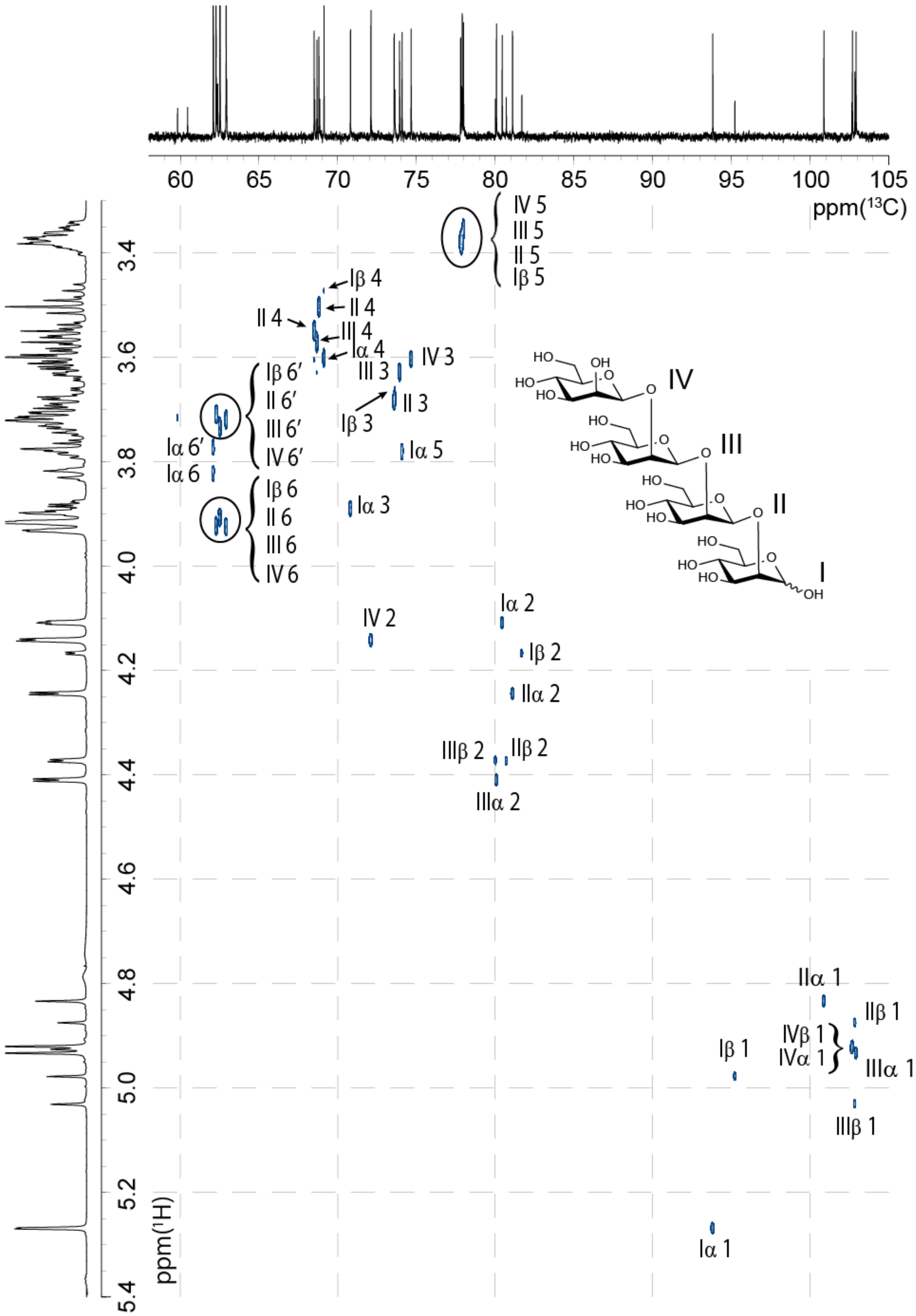

Figure S2. The NMR spectra of product 3 from the synthetic reaction catalyzed by Teth514_1788 with the substrates β-1,2-mannobiose and α-Man1*P*.

Product **3** was identified to be β-D-mannopyranosyl-(1→2)-β-D-mannopyranosyl-(1→2)-β-D-mannopyranosyl-(1→2)-D-mannose (β-1,2-Man4). The spectra were taken in D_2O, using 2-methyl-2-propanol as an internal standard (δ_H 1.23 and δ_C 31.2), using a Bruker DMX 800 spectrometer. The terms I, II, III, and IV on the spectra indicate reducing, two internal, and non-reducing D-mannose residues, respectively. The numbers after the letters indicate the positions on each sugar. (A) ^1H NMR spectrum; (B) ^{13}C NMR spectrum; (C) HSQC spectrum; and (D) HMBC spectrum.

(B) HMBC spectrum

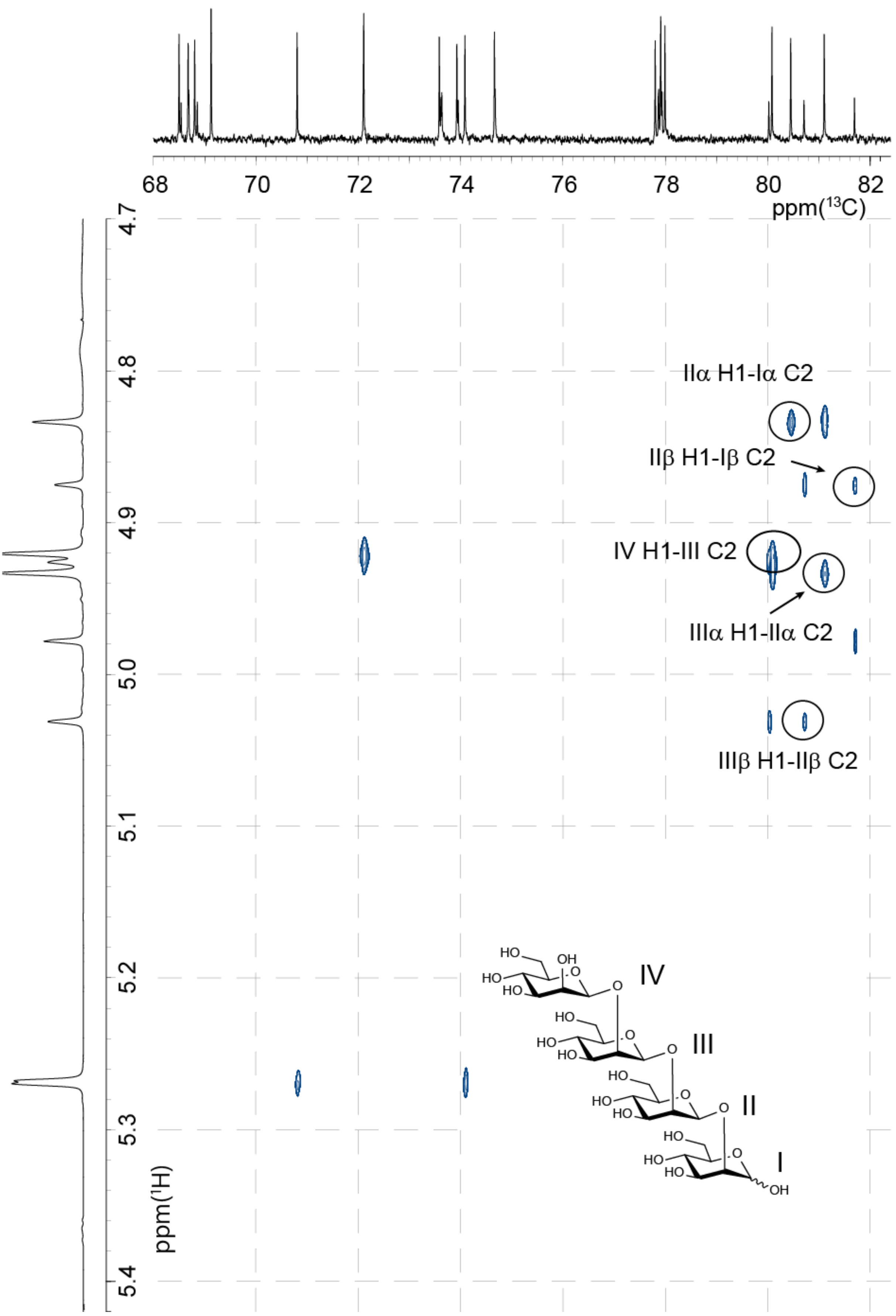

Figure S2-continud.
