# Supplementary material for: Discovery of Two β-1,2-Mannoside Phosphorylases Showing Different Chain-Length Specificities from Thermoanaerobacter sp. X-514
Source: PLoS One. 2014 Dec 12;9(12):e114882. doi: 10.1371/journal.pone.0114882 (PMC4264767; doi:10.1371/journal.pone.0114882)
Supplement: S3 Figure — A multiple alignment of the GH130 homologs. The multiple alignment was performed using ClustalW2 (http://www.ebi.ac.uk/Tools/msa/clustalw2/). BF0772, 4-O-β-d-mannosyl-d-glucose phosphorylase from Bacteroides fragilis (GenBank accession number CAH06518); Rumal_0852, 4-O-β-d-mannosyl-d-glucose phosphorylase from Ruminococcus albus (ADU21379); Rumal_0099, β-1,4-mannooligosaccharide phosphorylase from R. albus (ADU20661); BT_1033, β-1,4-d-mannosyl-N-acetyl-d-glucose phosphorylase from B. thetaiotaomicron (AAO76140); UhgbMP, β-mannopyranosyl-[N-glycan] phosphorylase from an uncultured human gut bacterium (ADD61463); TM1225, GH130 protein from Thermotoga maritime (AAD36300); BT_4094, GH130 protein from B. thetaiotaomicron (AAO79199); BDI_3141, GH130 protein from Parabacteroides distasonis (ABR44847); BACOVA_03624 and BACOVA_021614, GH130 proteins from B. ovatus (EDO10988 and EDO12271, respectively); Teth514_1788, 1,2-β-oligomannan phosphorylase from Thermoanaerobacter sp. X-514 (ABY93073); and Teth514_1789, β-1,2-mannobiose phosphorylase from Thermoanaerobacter sp. X-514 (ABY93074). Above the sequences, the secondary structures of BF0772 are shown as squiggles (α-helices), arrows (β-strands), and 'T' characters (β-turns). The strictly conserved residues are shown in a box with white characters. Residues that have similarity across the sequences are shown in a box with bold characters, indicating the similar residues in the sequence group. Black starred characters represent amino acid residues that are involved in the mannose recognition site of BF0772 (Asn73, Asp131, and Asp344). (PDF) [file pone.0114882.s003.pdf]

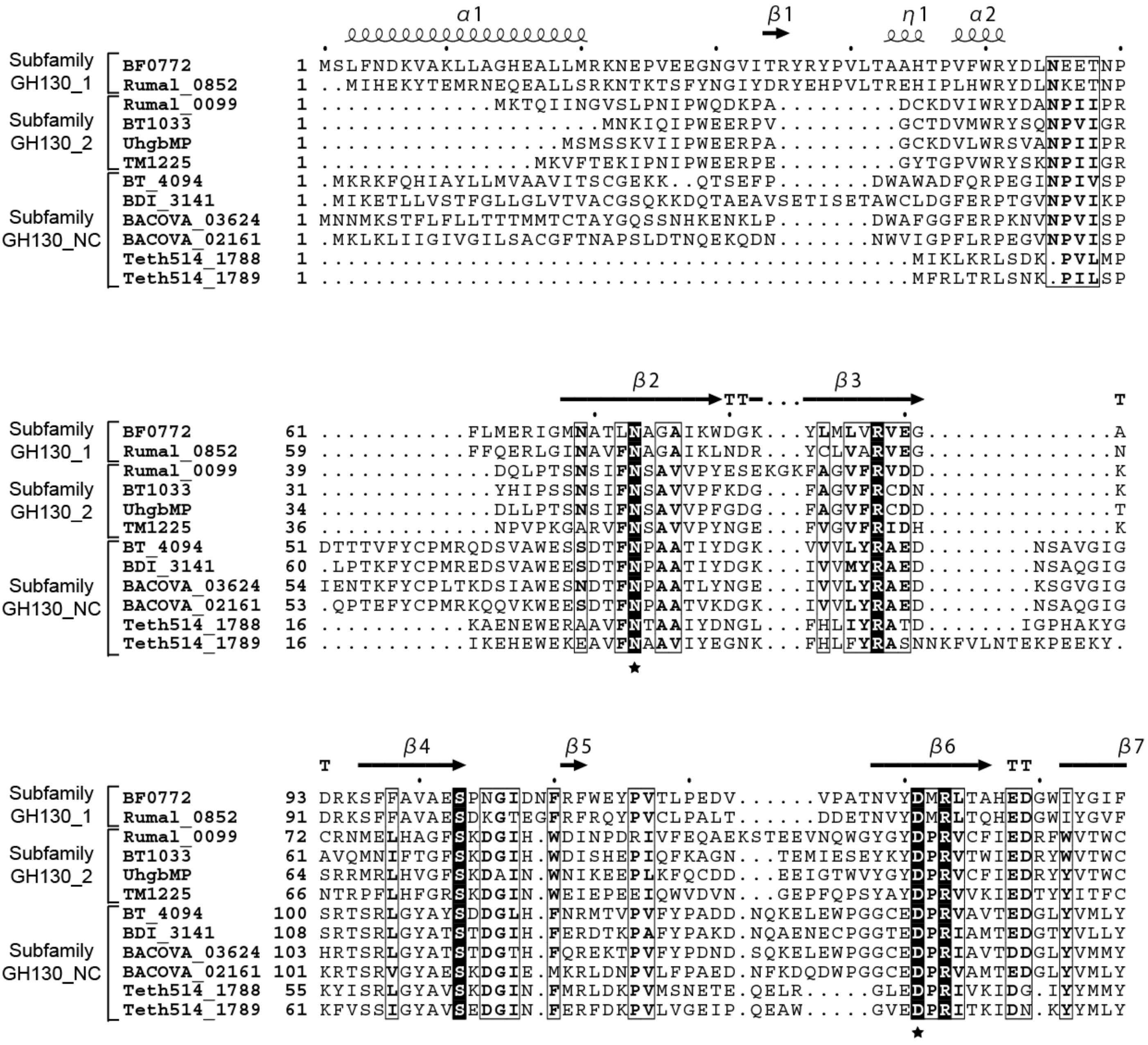

Figure S3. A Multiple alignment of the GH130 homologs.

The multiple alignment was performed using ClustalW2 (http://www.ebi.ac.uk/Tools/msa/clustalw2/). BF0772, 4-*O*-β-D-mannosyl-D-glucose phosphorylase from *Bacteroides fragilis* (GenBank[TM] accession number CAH06518); Rumal_0852, 4-*O*-β-D-mannosyl-D-glucose phosphorylase from *Ruminococcus albus* (ADU21379); Rumal_0099, β-1,4-mannooligosaccharide phosphorylase from *R. albus* (ADU20661); BT_1033, β-1,4-D-mannosyl-*N*-acetyl-D-glucose phosphorylase from *B. thetaiotaomicron* (AAO76140); UhgbMP, β-mannopyranosyl-[*N*-glycan] phosphorylase from an uncultured human gut bacterium (ADD61463); TM1225, GH130 protein from *Thermotoga maritime* (AAD36300); BT_4094, GH130 protein from *B. thetaiotaomicron* (AAO79199); BDI_3141, GH130 protein from *Parabacteroides distasonis* (ABR44847); BACOVA_03624 and BACOVA_021614, GH130 proteins from *B. ovatus* (EDO10988 and EDO12271, respectively); Teth514_1788 and Teth514_1789, 1,2-β-oligomannan phosphorylases from *Thermoanaerobacter* sp. X-514 (ABY93073, and ABY93074, respectively). Above the sequences, the secondary structures of BF0772 are shown as squiggles (α-helices), arrows (β-strands), and 'T' characters (β-turns). The strictly conserved residues are shown in a box with white characters. Residues that have similarity across the sequences are shown in a box with bold characters, indicating the similar residues in the sequence group. Black starred characters represent amino acid residues that are involved in the mannose recognition site of BF0772 (Asn73, Asp131, and Asp344).

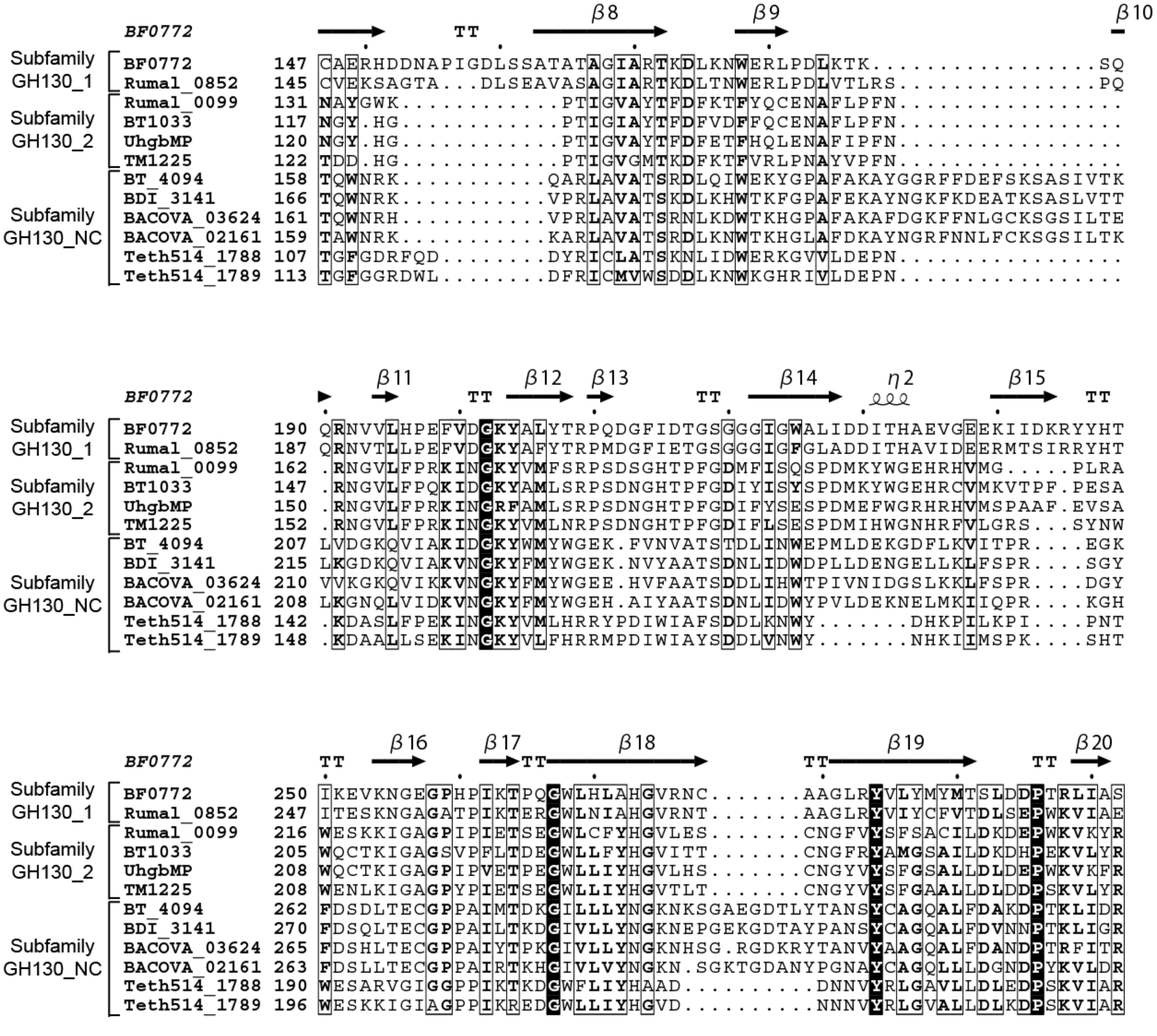

Figure S3-continued.

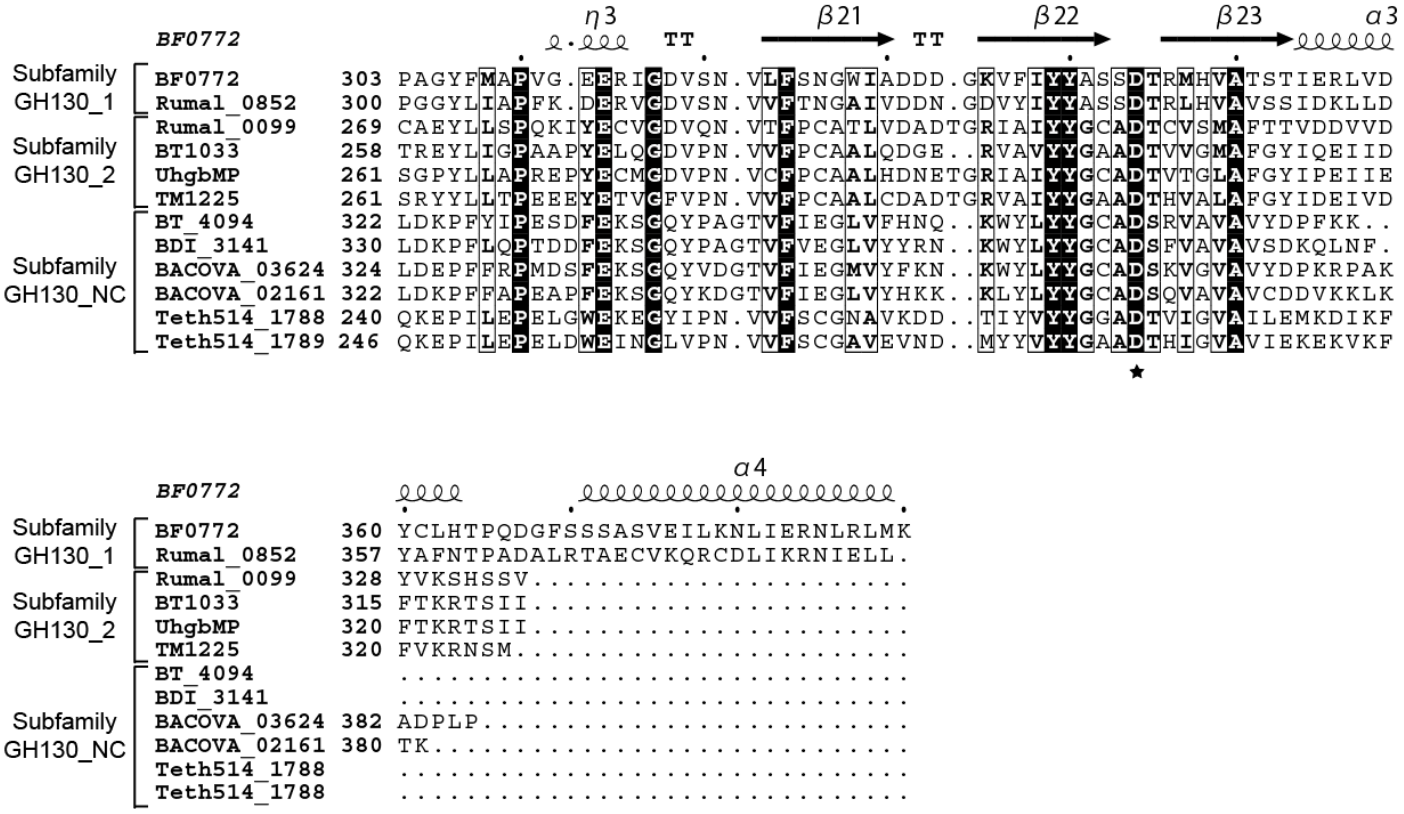

Figure S3-continued.
