# Supplementary material for: Discovery of Two β-1,2-Mannoside Phosphorylases Showing Different Chain-Length Specificities from Thermoanaerobacter sp. X-514
Source: PLoS One. 2014 Dec 12;9(12):e114882. doi: 10.1371/journal.pone.0114882 (PMC4264767; doi:10.1371/journal.pone.0114882)
Supplement: S1 Table — The chemical shifts in the 1H and 13C NMR spectra of the products of the synthetic reaction catalyzed by Teth514_1789 with the substrates d-mannose and α-Man1P. (PDF) [file pone.0114882.s004.pdf]

TABLE S1

The chemical shifts in the ^{1}H and ^{13}C NMR spectra of the products of the synthetic reaction catalyzed by Teth514_1789 with the substrates D-mannose and α-Man1*P*.

Sugar ring	Site	Product **1** α-Anomer (71%) ^{13}C δ (ppm)	α-Anomer ^{1}H δ (ppm)		α-Anomer *J* (Hz)	β-Anomer (29%) ^{13}C δ (ppm)	β-Anomer ^{1}H δ (ppm)		β-Anomer *J* (Hz)
Man I	1	93.5	5.26	d	$J_{1,2}$≈1	95.2	4.95	d	$J_{1,2}$≈1
	2	79.6	4.10	dd	$J_{2,3}$=2.9	81.0	4.15	dd	$J_{2,3}$=2.5
	3	71.2	3.83	m		74.0	3.60	m	
	4	68.8	3.67	m		68.4	3.62	m	
	5	74.1	3.78	m		77.9	3.35	m	
	6		3.82	m			3.89	m	
	6’	62.3	3.75	dd	$J_{5,6'}$=5.3 $J_{6,6'}$=11.6	62.5	3.71	m	
Man II	1	100.3	4.74	d	$J_{1,2}$≈1	102.5	4.80	d	$J_{1,2}$≈1
	2	72.3	4.01	dd	$J_{2,3}$=2.7	71.9	4.14	dd	$J_{2,3}$=2.5
	3	74.4	3.62	dd	$J_{3,4}$=9.6	73.7	3.65	m	
	4	68.4	3.54	dd	$J_{4,5}$=9.6	68.4	3.54	m	
	5	77.9	3.35	m		77.9	3.35	m	
	6		3.89	dd	$J_{5,6}$=1.9		3.89	m	
	6’	62.6	3.71	dd	$J_{5,6'}$=6.6 $J_{6,6'}$=12.3	62.6	3.71	m	

The spectra were taken in D_2O, using 2-methyl-2-propanol as an internal standard (δ_H 1.23 and δ_C 31.2), using a Bruker DMX 600 spectrometer. The terms Man I and Man II indicate the first and second D-mannose residues from the reducing end, respectively. Underlines represent HMBC correlations between the anomeric proton of Man II and the carbon of Man I, respectively.

TABLE S1-continued.

Sugar ring	Site	Product **2**							
		α-Anomer (72%)				β-Anomer (28%)			
		^{13}C δ (ppm)	^{1}H δ (ppm)		J (Hz)	^{13}C δ (ppm)	^{1}H δ (ppm)		J (Hz)
Man I	1	93.6	5.26	d	$J_{1,2}$=1.6	95.2	4.97	d	$J_{1,2}$≈1
	2	80.1	4.10	dd	$J_{2,3}$=2.8	79.7	4.40	dd	$J_{2,3}$=2.5
	3	70.8	3.88	m		73.7	3.65	m	$J_{3,4}$=9.8
	4	68.8	3.64	m		68.8	3.48	dd	$J_{4,5}$=9.8
	5	74.0	3.77	m		77.8	3.37	m	
	6	62.5	3.91	m		62.5	3.89	m	
	6’		3.72	m			3.71	m	
Man II	1	100.6	4.84	d	$J_{1,2}$≈1	102.6	4.90	d	$J_{1,2}$≈1
	2	80.1	4.26	dd	$J_{2,3}$=2.7	81.2	4.15	dd	$J_{2,3}$=2.7
	3	73.8	3.65	m		74.0	3.62	m	
	4	68.6	3.60	m		68.6	3.60	m	
	5	77.8	3.37	m		77.8	3.37	m	
	6	62.6	3.91	m		62.6	3.89	m	
	6’		3.72	m			3.71	m	
Man III	1	102.5	4.85	d	$J_{1,2}$≈1	102.4	4.94	d	$J_{1,2}$≈1
	2	72.0	4.15	m		72.1	4.13	m	
	3	74.5	3.61	m		74.5	3.61	m	
	4	68.4	3.55	m		68.4	3.55	m	
	5	77.9	3.37	m		77.9	3.37	m	
	6	62.6	3.91	m		62.6	3.91	m	
	6’		3.72	m			3.72	m	

The spectra were taken in D_2O, using 2-methyl-2-propanol as an internal standard (δ_H 1.23 and δ_C 31.2), using a Bruker DMX 600 spectrometer. The terms Man I, Man II, and Man III indicate the first, second, and third D-mannose residues from the reducing end, respectively. Underlines represent HMBC correlations between the anomeric proton of Man II and the carbon of Man I and between the anomeric proton of Man III and the carbon of Man II, respectively.
