# Supplementary material for: Discovery of Two β-1,2-Mannoside Phosphorylases Showing Different Chain-Length Specificities from Thermoanaerobacter sp. X-514
Source: PLoS One. 2014 Dec 12;9(12):e114882. doi: 10.1371/journal.pone.0114882 (PMC4264767; doi:10.1371/journal.pone.0114882)
Supplement: S2 Table — The chemical shifts in the 1H and 13C NMR spectra of the products of the synthetic reaction catalyzed by Teth514_1789 with the substrates d-fructose and α-Man1P. (PDF) [file pone.0114882.s005.pdf]

TABLE S2

The chemical shifts in the ^{1}H and ^{13}C NMR spectra of the products of the synthetic reaction catalyzed by Teth514_1789 with the substrates D-fructose and α-Man1*P*.

		Product **4**				Product **5**			
Sugar ring	Site	^{13}C δ (ppm)	^{1}H δ (ppm)	*J* (Hz)		^{13}C δ (ppm)	^{1}H δ (ppm)	*J* (Hz)	
Fru	1	65.3	3.71	d	$J_{1,1'}$=11.7	65.0	3.70	d	$J_{1,1'}$=11.7
	1’		3.53	d			3.48	d	
	2	99.8	–			99.8	–		
	3	69.5	3.84	d	$J_{3,4}$=10.3	69.3	3.79	d	$J_{3,4}$=10.2
	4	70.7	3.92	m		70.4	3.94	m	
	5	77.8	4.21	m		78.4	4.18	m	
	6	62.2	3.96	dd	$J_{6,6'}$=13.0	62.5	3.91	m	$J_{6,6'}$=13.2
	6’		3.86	dd	$J_{5,6'}$=1.3		3.83	dd	$J_{5,6'}$=1.5
Man I	1	99.3	4.79	d	$J_{1,2}$≈1	99.6	4.85	d	$J_{1,2}$≈1
	2	72.6	4.05	dd	$J_{2,3}$=3.3	80.3	4.27	dd	$J_{2,3}$=3.1
	3	74.5	3.66	dd	$J_{3,4}$=9.6	73.8	3.67	dd	$J_{3,4}$=9.8
	4	68.5	3.57	dd	$J_{4,5}$=9.6	68.6	3.60	dd	$J_{4,5}$=9.8
	5	78.0	3.37	ddd	$J_{5,6}$=2.0	77.9	3.38	ddd	$J_{5,6}$=2.1
	6	62.7	3.91	dd	$J_{6,6'}$=12.4	62.5	3.91	m	
	6’		3.72	dd	$J_{5,6'}$=6.6		3.73	m	$J_{5,6'}$=6.2
Man II	1	–	–			102.6	4.86	d	$J_{1,2}$≈1
	2	–	–			71.9	4.17	dd	$J_{2,3}$=3.2
	3	–	–			74.4	3.62	dd	$J_{3,4}$=9.6
	4	–	–			68.3	3.55	dd	$J_{4,5}$=9.6
	5	–	–			77.9	3.34	ddd	$J_{5,6}$=1.9
	6	–	–			62.5	3.91	m	
	6’		–				3.73	m	$J_{5,6'}$=6.7

The spectra were taken in D_2O, using 2-methyl-2-propanol as an internal standard (δ_H 1.23 and δ_C 31.2), using a Bruker DMX 600 spectrometer. The terms Fru, Man I, and Man II indicate the first β-D-fructopyranose residue and second and third D-mannosyl residues from the reducing end, respectively. In the ^{1}H and ^{13}C NMR spectroscopies of products **4** and **5**, liner form of D-fructose and α-D-fructopyranose were not detected. Underlines represent HMBC correlations between the anomeric proton of Man I and the carbons of Fru and between the anomeric protons of Man II and the carbons of Man I, respectively.
