# Supplementary material for: Discovery of Two β-1,2-Mannoside Phosphorylases Showing Different Chain-Length Specificities from Thermoanaerobacter sp. X-514
Source: PLoS One. 2014 Dec 12;9(12):e114882. doi: 10.1371/journal.pone.0114882 (PMC4264767; doi:10.1371/journal.pone.0114882)
Supplement: S3 Table — The deduced amino acid sequence similarities of the genes involved in GDP-d-mannose biosynthesis in Thermoanaerobacter sp. X-514. The similarities between the amino acid sequences were investigated using the BLASTP program (Swiss-Prot/TrEMBL database). (PDF) [file pone.0114882.s006.pdf]

**Table S3. The deduced amino acid sequence similarities of the genes involved in GDP-D-mannose biosynthesis in *Thermoanaerobacter* sp. X-514. The similarities between the amino acid sequences were investigated using the BLASTP program (Swiss-Prot/TrEMBL database)**

| | | Accession no. | Score | Identity (%) | Similarity (%) | Gap (%) | Ref. | Function |
|---|---|---|---|---|---|---|---|---|
| Teth514_1796 | *Thermoanaerobacter pseudethanolicus* ATCC 33223 extracellular solute-binding protein | B0K7Y9 | 1264 | 100 | 100 | 0 | 1 | Extracellular solute-binding protein |
| | *Thermoanaerobacter thermohydrosulfuricus* ABC-type dipeptide transport system, periplasmic component | M8DNC8 | 1256 | 99 | 99 | 0 | 2 | |
| | *Haemophilus influenzae* Rd KW20 binding protein HI_0213; Flags: Precursor | P44572 | 58.2 | 24 | 39 | 24 | 2 | |
| | *Bacillus subtilis* subsp. *subtilis* str. 168 oligopeptide-binding protein AppA | P42061 | 42.7 | 24 | 40 | 20 | 3 | |
| Teth514_1795 | *Thermoanaerobacter pseudethanolicus* ATCC 33223 binding-protein-dependent transport system inner membrane protein | B0K7Y8 | 652 | 100 | 100 | 0 | 4 | Oligosaccharide transport system permease protein |
| | *Thermoanaerobacter thermohydrosulfuricus* ABC-type dipeptide/oligopeptide/nickel transport systems, permease components | M8CLH7 | 649 | 99 | 100 | 0 | 5 | |
| | *Sinorhizobium fredii* NGR234 peptide ABC transporter permease protein y4tP | Q53191 | 144 | 28 | 54 | 5 | 6 | |
| | *Bacillus subtilis* subsp. *subtilis* str. 168 oligopeptide transport system permease protein AppB | P42062 | 136 | 28 | 51 | 4 | 3 | |
| Teth514_1794 | *Thermoanaerobacter pseudethanolicus* ATCC 33223 binding-protein-dependent transport system inner membrane protein | B0K7Y7 | 581 | 100 | 100 | 0 | 7 | Oligosaccharide transport system permease protein |
| | *Thermoanaerobacter thermohydrosulfuricus* ABC-type dipeptide/oligopeptide/nickel transport systems, permease components | M8DDH8 | 570 | 97 | 99 | 0 | 5 | |
| | *Bacillus subtilis* subsp. *subtilis* str. 168 oligopeptide transport system permease protein AppC | P42063 | 103 | 26 | 48 | 5 | 3 | |
| | *Bacillus subtilis* subsp. *subtilis* str. 168 dipeptide transport system permease protein DppC | P94312 | 102 | 28 | 51 | 4 | 3 | |
| Teth514_1793 | *Thermoanaerobacter pseudethanolicus* ATCC 33223 oligopeptide/dipeptide ABC transporter ATPase | B0K7Y6 | 662 | 100 | 100 | 0 | 8 | ATP-binding protein |
| | *Thermoanaerobacter thermohydrosulfuricus* oligopeptide/dipeptide ABC transporter, ATP-binding protein | M8CLI2 | 656 | 99 | 99 | 0 | 5 | |
| | *Bacillus subtilis* subsp. *subtilis* str. 168 dipeptide transport ATP-binding protein DppD | P26905 | 248 | 37 | 60 | 3 | 3 | |
| | *Bacillus subtilis* subsp. *subtilis* str. 168 oligopeptide transport ATP-binding protein AppD | P42064 | 245 | 38 | 60 | 0 | 3 | |
| Teth514_1792 | *Thermoanaerobacter pseudethanolicus* ATCC 33223 oligopeptide/dipeptide ABC transporter ATPase | B0K7Y5 | 669 | 100 | 100 | 0 | 9 | ATP-binding protein |
| | *Thermoanaerobacter thermohydrosulfuricus* oligopeptide/dipeptide ABC transporter, ATP-binding protein | M8CUP7 | 664 | 99 | 99 | 0 | 5 | |
| | *Haemophilus influenzae* Rd KW20 dipeptide transport ATP-binding protein DppF | P45094 | 225 | 39 | 59 | 2 | 2 | |
| | *Sinorhizobium fredii* NGR234 peptide ABC transporter ATP-binding protein y4tS | Q53194 | 224 | 38 | 58 | 3 | 10 | |
| Teth514_1791 | *Thermoanaerobacter italicus* Ab9 hypothetical protein | D3T579 | 101 | 98 | 98 | 0 | 11 | Hypothetical protein |
| | *Caldicellulosiruptor saccharolyticus* DSM 8903 hypothetical protein | A4XG78 | 50.4 | 47 | 74 | 1 | 12 | |
| | *Caldicellulosiruptor hydrothermalis* 108 hypothetical protein | E4Q8C1 | 42.4 | 42 | 67 | 0 | 13 | |
| Teth514_1790 | *Thermoanaerobacter pseudethanolicus* ATCC 33223 glycoside hydrolase family protein | B0K7Y4 | 1295 | 100 | 100 | 0 | 14 | GH5 β-glycoside hydrolase |
| | *Thermoanaerobacter wiegelii* Rt8.B1 β-galactosidase trimerisation domain-containing protein | G2MUR1 | 1279 | 98 | 99 | 0 | 15 | |
| | *Aspergillus niger* CBS 513.88 mannan *endo*-1,4-β-mannosidase A | A2QKT4 | 55.8 | 29 | 43 | 16 | 16 | |
| | *Aspergillus oryzae* RIB40 mannan *endo*-1,4-β-mannosidase A | Q2TXJ2 | 52.4 | 23 | 37 | 16 | 17 | |
| Teth514_1789 | *Thermoanaerobacter pseudethanolicus* ATCC 33223 glycosidase | B0K7Y3 | 618 | 100 | 100 | 0 | 18 | GH130 phosphorylase |
| | *Thermoanaerobacter ethanolicus* glycosidase-like protein | G2MUR2 | 597 | 96 | 98 | 0 | 19 | |
| | *Ruminococcus albus* 7 β-1,4-mannooligosaccharide phosphorylase | E6UBR9 | 114 | 31 | 47 | 8 | 20 | |
| | *Ruminococcus albus* 7 4-*O*-β-D-mannosyl-D-glucose phosphorylase | E6UIS7 | 82.0 | 27 | 43 | 12 | 20 | |
| Teth514_1788 | *Thermoanaerobacter pseudethanolicus* ATCC 33223 glycosidase | B0K7Y2 | 606 | 100 | 100 | 0 | 21 | GH130 phosphorylase |
| | *Thermoanaerobacter wiegelii* Rt8.B1 glycosidase related protein | G2MUR3 | 591 | 97 | 98 | 0 | 22 | |
| | *Ruminococcus albus* 7 β-1,4-mannooligosaccharide phosphorylase | E6UBR9 | 122 | 30 | 48 | 14 | 20 | |
| | *Ruminococcus albus* 7 4-*O*-β-D-mannosyl-D-glucose phosphorylase | E6UIS7 | 71.6 | 28 | 43 | 12 | 20 | |
| Teth514_1787 | *Thermoanaerobacter pseudethanolicus* ATCC 33223 nucleotidyl transferase | B0K7Y1 | 705 | 99 | 99 | 0 | 23 | Mannose-1-phosphate guanylyltransferase |
| | *Thermoanaerobacter ethanolicus* nucleotidyltransferase | F1ZZ53 | 695 | 97 | 99 | 0 | 24 | |
| | *Caenorhabditis briggsae* mannose-1-phosphate guanyltransferase β | Q61S97 | 192 | 34 | 53 | 5 | 25 | |
| | *Caenorhabditis elegans* mannose-1-phosphate guanyltransferase β | A3QMC8 | 187 | 34 | 54 | 5 | 26 | |
| Teth514_1786 | *Thermoanaerobacter pseudethanolicus* ATCC 33223 group 1 glycosyl transferase | B0K7Y0 | 788 | 99 | 99 | 0 | 27 | GT4 GDP-mannose-dependent α-mannosyltransferase |
| | *Thermoanaerobacter wiegelii* Rt8.B1 group 1 glycosyl transferase | G2MUR5 | 772 | 97 | 98 | 0 | 28 | |
| | *Mycobacterium smegmatis* str. MC2 155 GDP-mannose-dependent α-mannosyltransferase | A0R043 | 58.9 | 23 | 39 | 9 | 29 | |
| | *Mycobacterium tuberculosis* GDP-mannose-dependent α-mannosyltransferase | O53522 | 48.5 | 20 | 40 | 11 | 30 | |
| Teth514_1785 | *Thermoanaerobacter pseudethanolicus* ATCC 33223 hypothetical protein | B0K7X9 | 699 | 98 | 98 | 0 | 31 | Hypothetical protein |
| | *Thermoanaerobacter wiegelii* Rt8.B1 hypothetical protein | G2MUR6 | 674 | 93 | 96 | 0 | 32 | |
| | *Thermoanaerobacter siderophilus* glycosyltransferase | I9KTU4 | 674 | 93 | 96 | 0 | 33 | |
| | *Caldanaerobacter subterraneus* glycosyltransferase | U5CN06 | 512 | 69 | 84 | 0 | 34 | |
| | *Clostridium stercorarium* subsp. *stercorarium* DSM 8532 glycosyltransferase | L7VKB4 | 373 | 51 | 73 | 2 | 35 | |

1. Copeland, A. *et al*., (2008) GenBank (ABY95809.1); 2. Fleischmann, R. D. *et al*., (1995) *Science* **269**, 496-512; 3. Mathiopoulos, C., *et al*., (1991) *Mol. Microbiol.* **5**, 1903-1913; 4. Copeland, A. *et al*., (2008) GenBank (ABY95808.1); 5. Verbeke, T. J. *et al*., (2013) *PLoS One* **8**, e59362; 6. Schmeisser, C. *et al*., (2009) *Appl. Environ. Microbiol.* **75**, 4035-4045; 7. Copeland, A. *et al*., (2008) GenBank (ABY95807.1); 8. Copeland, A. *et al*., (2008) GenBank (ABY95806.1); 9. Copeland, A. *et al*., (2008) GenBank (ABY95805.1); 10. Freiberg, C. *et al*., (1996) *Genome Res.* **6**, 590-600; 11. Lucas, S. *et al*., (2011) GenBank (ADD01380.1); 12. Copeland, A. *et al*., (2007) GenBank (ABP65913.1); 13. Blumer-Schuette, S. E. *et al*., (2011) *J. Bacteriol.* **193**, 1483-1484; 14. Copeland, A. *et al*., (2008) GenBank (ABY95804.1); 15. Lucas, S. *et al*., (2011) GenBank (AEM77584.1); 16. Pel, H. J. *et al*., (2007) *Nature Biotechnol.* **25**, 221-231; 17. Machida, M. *et al*., (2005) *Nature* **438**, 1157-1161; 18. Copeland, A. *et al*., (2008) GenBank (ABY95803.1); 19. Lucas, S. *et al*., (2011) GenBank (AEM77585.1); 20. Kawahara, R. *et al*., (2012) *J. Biol. Chem.* **287**, 42389-42399; 21. Copeland, A. *et al*., (2008) GenBank (ABY95802.1); 22. Lucas, S. *et al*., (2011) GenBank (AEM77586.1); 23. Copeland, A. *et al*., (2008) GenBank (ABY95801.1); 24. Lucas, S. *et al*., (2011) GenBank (EGD50599.1); 25. Stein, L. D. *et al*., (2003) *PLoS Biology* **1**, E45; 26. Consortium, C. e. S. *et al*., (1998) *Science* **282**, 2012-2018; 27. Copeland, A. *et al*., (2008) GenBank (ABY95800.1); 28. Lucas, S. *et al*., (2011) GenBank (AEM77588.1); 29. Guerin, M. E. *et al*., (2009) *J. Biol. Chem.* **284**, 25687-25696; 30. Torrelles, J. B. *et al*., (2009) *Glycobiology* **19**, 743-755; 31. Copeland, A. *et al*., (2008) GenBank (ABY95799.1); 32. Lucas, S. *et al*., (2011) GenBank (AEM77589.1); 33. Lucas, S. *et al*. (2012) GenBank (EIW00286.1); 34. Lee, S. J. *et al*., (2013) *Genome announcements* **1**, 10.1128/genomeA.00923-00913; 35. Poehlein, A. *et al*., (2013) GenBank (AGC67099.1).
